# Internalisation of environmental costs of decentralised nitrogen fertilisers production

**DOI:** 10.1007/s11367-023-02187-5

**Published:** 2023-06-09

**Authors:** Jose Luis Osorio-Tejada, Evgeny Rebrov, Volker Hessel

**Affiliations:** 1 School of Engineering, University of Warwick, Coventry, CV4 7AL UK; 2 School of Chemical Engineering, University of Adelaide, Adelaide, SA 5005 Australia; 3 Faculty of Environmental Sciences, Universidad Tecnológica de Pereira, Pereira, Colombia

**Keywords:** Plasma, LCA, Fertilisers, Ammonia, External costs, Supply chain, Local production

## Abstract

**Purpose:**

Ammonia (NH_3_) production is an energy-intensive process that is concentrated in a few countries at large-scale plants, mainly using the Haber–Bosch (HB) process. Local plants next to farmers can reduce environmental impacts, as well as reduce storage, shortage risks, and price volatility of fertilisers. Since local NH_3_ production is not cost-effective, we analyse how internalisation of environmental impacts into economic analyses could help to promote novel technologies for NH_3_ synthesis when supplied with renewable energy.

**Methods:**

Mini-HB plants working at high pressure and temperature, as well as novel alternatives based on plasma reactors working at ambient conditions and using electricity from renewable sources, have been recently proposed for decentralised NH_3_ production. To evaluate the environmental performances of these alternative and traditional NH_3_ pathways, a life cycle assessment was performed to quantify the reduced emissions in each production process and the impacts of by-product utilisation, such as steam, oxygen, or carbon black. Different scales of storage and transportation, fuelled by traditional energy sources, were modelled to quantify the impacts of the simplified NH_3_ supply chains. A review of monetary valuation coefficients was performed to internalise the life cycle environmental impacts into the techno-economic analyses of NH_3_ production in Australia.

**Results and discussion:**

Most of the estimated environmental costs were due to the carbon emissions of conventional plants and thermal plasma plants because of the use of fossil-based electricity. However, the high external costs associated with the photochemical oxidant formation and particulate matter affected the thermal plasma and non-thermal plasma (NTP) plants, costing in total 9,500 and 4,200 $/t NH_3,_ respectively, due to the impacts of solar panels manufacturing. In contrast, electrolyser-HB plants obtained rates of 114 $/t NH_3_ because of the high energy efficiency and oxygen sales. In the future scenario for NTP-based plants, this alternative could also be competitive with rates of 222 $/t NH_3_. Additionally, the estimated total external costs for the conventional NH_3_ industry in Australia amounted to about US$5 billion per year.

**Conclusions:**

Electrolyser-HB plants could be cost-effective in the short term due to the energy efficiency of HB processes. However, the HB process has reached its efficiency limits, while the NTP process still has room for improvement, as well as its production costs are lower at smaller scales. In addition, if monetised environmental costs are analysed for a whole industry, public administrations could be prompted to invest the expected savings in the promotion of these novel technologies.

**Supplementary Information:**

The online version contains supplementary material available at 10.1007/s11367-023-02187-5.

## Introduction

Ammonia (NH_3_) is an indispensable fertiliser feedstock to sustain global food production. Over 90% of NH_3_ is produced from hydrogen (H_2_) and nitrogen (N_2_) through the Haber–Bosch (HB) process (Fúnez-Guerra et al. 2020)), mainly using natural gas (NG) via the steam methane reforming (SMR) process (Parkinson et al. 2018). This energy-intensive process has a carbon footprint of about 1.6 t CO_2_/t NH_3_, contributing to 1.8% of global carbon emissions (The Royal Society 2020).

Dependency on energy feedstock has led to the concentration of NH_3_ production in a few countries where non-expensive NG is available, such as Trinidad and Tobago, Russia, Saudi Arabia, and the United States, or in countries with abundant coal reserves, such as China (IEA 2021; Indexbox 2023). This centralisation of NH_3_ production increases the carbon footprint of fertilisers when exported to different continents. Moreover, the volatility of NG prices has revealed the unsustainability of conventional fertilisers production. The rise in NG prices by the end of 2021 increased the cost of each NH_3_ tonne in Europe from US$ 300 up to US$ 810 (Durisin 2021). The recent trade disruptions related to the pandemic lockdowns have also increased international shipping costs and time. Additionally, the NH_3_ offer curtailment due to the Russia-Ukraine conflict has escalated NH_3_ prices in March 2022 reaching peaks of US$ 1,625 in Tampa (Agroberichten Buitenland 2022). Food security in countries with a high dependency on imported fertilisers has been affected to a greater extent. For example, in Peru, 68.5%, 97.4%, and 50.9% of NH_3_-derived fertilisers such as urea, ammonium nitrate, and ammonium sulphate, were imported from Russia in 2021, respectively (Government of Peru 2022). These factors have generated a local shortage of fertilisers and tripled their prices, triggering protests of farmers and inflation because fertilisers usually share about 27% of the total production costs of basic food products such as rice (Agencia Agraria de Noticias 2022; Adepia 2022). Furthermore, challenges encountered in inland transportation significantly increase the cost of fertilisers for end-users, particularly in landlocked countries and regions with rough topography and limited rail transport. In these areas, the distribution of fertilisers heavily relies on expensive road transport, especially in South America and Sub-Saharan Africa (Roberts and Vilakazi 2014; Vilakazi 2018; IFDC 2019; Takele and Tolcha 2021). Similarly, countries with geographically dispersed agricultural areas away from cities and ports, such as Australia, encounter high costs for inland distribution of fertilisers due to the limited railway network connections near end-users (Tran et al. 2021).

These drawbacks have made us aware of the need to move towards an economic model based on a resilient and self-sufficient decentralised production. Small-scale NH_3_ distributed plants next to farmers, besides mitigating the global supply chain issues, can create additional benefits such as local employment, the utilization of local resources, technological and knowledge transfer, crop intensification by a tailored fertiliser production, and environmental benefits due to the use of cleaner energy sources.

Different plant configurations have been proposed to produce cleaner NH_3_ than the conventional NG-based plants which produce “grey NH_3_”, such as the “turquoise NH_3_” plants that also use NG but through high-temperature plasma (HTP) pyrolysis for the H_2_ production which does not release CO_2_ (Long et al. 2021; Sarafraz et al. 2021; Osorio-Tejada et al. 2022c), or “green NH_3_” plants that use electricity from renewable sources to produce H_2_ through water electrolysis to be used in all-electric HB (eHB) plants (Morgan 2013; Fasihi et al. 2021) or in novel non-thermal plasma (NTP) reactors (Anastasopoulou et al. 2020b; Osorio-Tejada et al. 2022b). NTP systems have been recently proposed as promissory alternative because they can be turned off and on quickly due to low thermal inertia (Snoeckx and Bogaerts 2017), in contrast to the HB systems, which ideally run continuously, that is, they take many hours to reach a steady state process (Muelaner 2020), which does not interface with the intermittency of renewable energies.

The feasibility of these emerging technologies can be promoted by the internalisation of the environmental benefits. Fasihi et al. (2021) analysed NH_3_ production costs at factory gate of electrolyser-eHB plants in different locations supplied by photovoltaic and wind electricity. They concluded that these plants would be cost-effective by 2040 if a carbon tax of 75 €/t CO_2_ were charged. However, they assumed that renewable-based electricity was zero emissions, omitting the life cycle emissions of these resources and other environmental impacts. In a recent study (Osorio-Tejada et al. 2022b), we performed life cycle assessments (LCA) for conventional and alternative NH_3_ production pathways considering relevant environmental impact categories in chemicals production (Maranghi and Brondi 2020; Osorio-Tejada et al. 2022a), concluding that the electrolyser-HB plants fed with solar energy can generate only 0.12 t CO_2_/t NH_3,_ in comparison to 2.10 t CO_2_/t NH_3_ of conventional plants, in a cradle-to-site (farm) assessment. These alternatives for distributed production also reduced ozone formation impacts caused by NOx emitted by diesel trucks, as well as reduced water eutrophication impacts caused by the fossil-based electricity from the national grid, especially from the hard coal and lignite power plants. However, these small-scale plants for distributed NH_3_ production cannot reach the economics of the large-scale centralised NH_3_ plants, costing around three times higher than the average (Tran et al. 2021). For this reason, despite the benefits of local production, alternative plants have not been widely deployed. Even if taxes on carbon emission are imposed, alternative plants would not be competitive during periods of low NG prices.

In that regard, to enhance the competitiveness of cleaner production processes, there have been proposals to internalize emissions other than CO_2_ through various methods aimed at monetising environmental impacts based on LCA impact categories. Monetisation represents the weight of an impact category in monetary value based on the costs of preventing or repairing damages, as well as how much society is willing to pay to prevent these impacts (Durão et al. 2019). However, hitherto there is no consensus in the scientific community on the most suitable monetisation method (Thi et al. 2016; Canaj et al. 2021). This is because of the high uncertainty in estimating monetary valuation coefficients (MVC) due to diverse factors involved, including: *(i)* the monetisation approach, which can be based on abatement costs, budget constraints, damage costs or societal willingness-to-pay (Pizzol et al. 2015; Arendt et al. 2020); *(ii)* the geography, as methods developed in Europe might not be applicable to other continents due to different market prices and budget constraints, resulting in very different MVC (Amadei et al. 2021); and *(iii)* the followed life cycle impacts assessment method, which can be based on a few impact categories (Davidson et al. 2005; Ahlroth and Finnveden 2011; European Commission et al. 2019), or several impact categories, such as the 16 categories of the Product Environmental Footprint method (European Commission 2018) utilised in (European Commission et al. 2020; Amadei et al. 2021), or the 18 categories of the ReCiPe 2008 (Goedkoop et al. 2009) and 2016 (Huijbregts et al. 2017) methods utilised in (de Bruyn et al. 2018) and (Ponsioen et al. 2020).

In a recent review on monetisation methods in LCA, Amadei et al. (2021) analysed 18 methods that presented MVC for diverse impact categories. Many of these methods considered different lists of impact categories and some had similar impact categories but with different units (e.g., ionizing radiation can be expressed in either kg U-235 eq. or kg Co-60 eq.), making them incomparable. Only six impact categories were common across diverse methods by using the same units and monetisation approach (e.g., damage cost), These categories were climate change, ozone depletion, particulate matter, ionizing radiation, photochemical ozone formation, and acidification. Although the monetisation methods utilise a similar approach, the uncertainty surrounding the choice of a specific method is exemplified by the fact that the MVC for these six impact categories can vary by up to three orders of magnitude (Amadei et al. 2021). This level of uncertainty would suggest the need for sensitivity analysis or comparison of different monetisation methods when no specific method is identified as appropriate for a given study.

In brief, we perform a techno-economic analysis (TEA) and a LCA of different NH_3_ production pathways to estimate the cost and environmental impacts of NH_3_ at farm in Australia. In this country, most of the nitrogen fertilisers are imported and the domestic production is concentrated in large-scale plants located near ports, resulting in long-distance transport of fertilisers to inner agriculture areas (Fertilizers Australia 2019; Tran et al. 2021). In this sense, the TEA and LCA consider credits of by-products utilisation and reduced emissions in the production stage by using novel technologies, as well as the impacts of storage and transport. Moreover, LCA results are monetised and internalised using MVC for five impact categories to identify the most cost-effective configuration when environmental aspects other than carbon credits are considered.

## Methods

The environmental profiles of the different NH_3_ production pathways were estimated according to the standard ISO 14044 (ISO 2006) for LCA studies. The TEA was performed by estimating the unitary cost of production (UCOP) of NH_3_, as well as the cost of its storage and transport to provide insights into the economic performance of the conventional and alternative pathways. We estimated the external costs of NH_3_ production, that is, the costs that society would pay to mitigate the damages to human health, ecosystems and material resources caused by the emissions in the NH_3_ supply chain, which are not generally assumed by producers. Finally, we internalise the estimated external costs in the economic analysis of each NH_3_ supply chain.

The UCOP of NH_3_ was obtained after estimating the annual operating costs (Opex) and the annualized capital cost (ACC) (Towler and Sinnott 2013). The ACC was calculated by considering an annual capital charge ratio (ACCR) with a 10% interest rate (*i*) and a 15-year lifespan *(n)*, as shown in Eqs. (1–3)*.* Opex comprises both annual variable operating costs, which correspond to the actual cost of feedstocks and energy inputs and outputs (by-products), and annual fixed operating costs, such as labour, maintenance, insurance, property taxes, and other related expenses. The fixed operating costs were estimated using a factorial approach linked to the total capital costs (Capex), which represent the actual non-installed equipment cost plus the cost of installation (Peters et al. 2003).

1 $$UCOP=\frac{Opex+ACC}{annual\ plant\ capacity}$$

2 $$ACC=ACCR\times Capex$$

3 $$ACCR=\frac{[{\mathrm{i}\left(1+\mathrm{i}\right)}^{\mathrm{n}}]}{{[\left(1+\mathrm{i}\right)}^{\mathrm{n}}-1]}$$

### Goal and scope definition

**Fig. 1:**
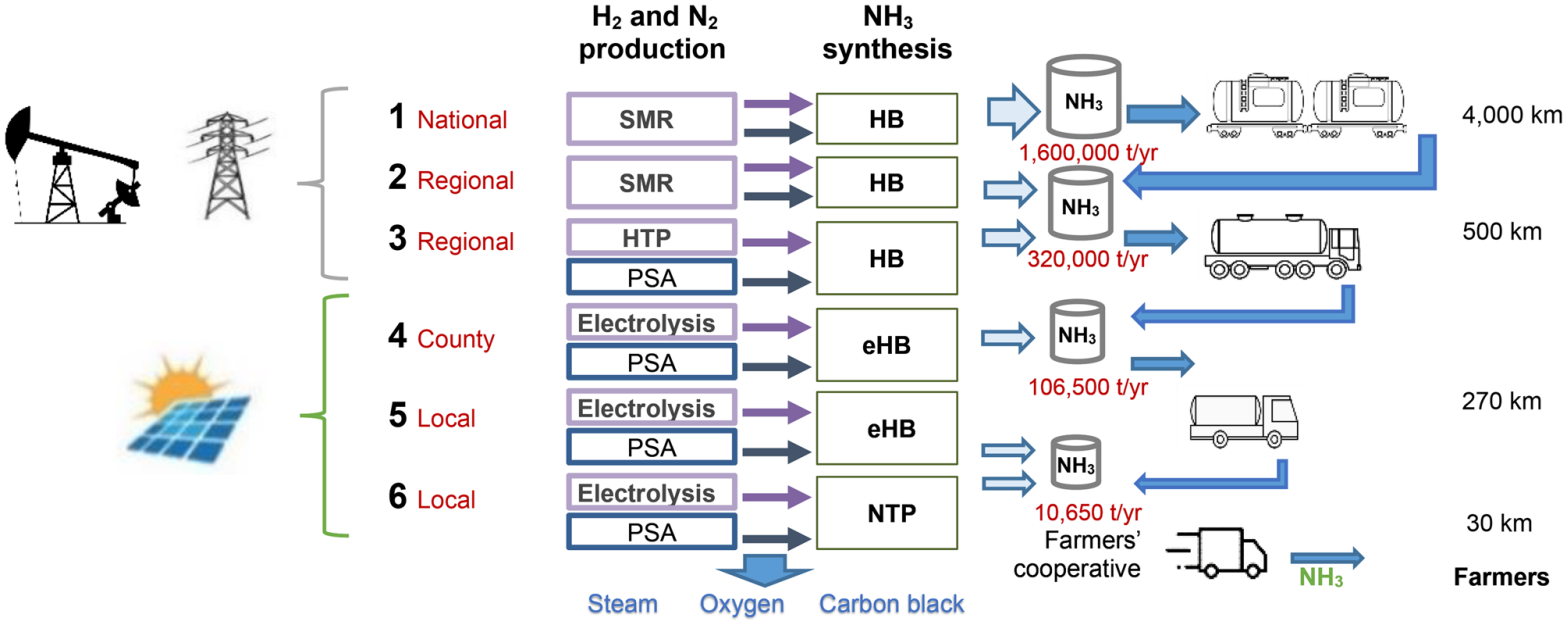
NH_3_ production pathways. Note: PSA stands for Pressure Swing Absorption unit for the nitrogen production. In the conventional pathway 1, nitrogen production is included in the SMR process

This study aims to analyse the extent to which cleaner alternative NH_3_ production pathways can be cost-effective when environmental benefits are internalised in the economics of the supply chain in a cradle-to-site evaluation in Australia, in comparison to the supply chain of NH_3_ produced at conventional large-scale HB plants based on SMR process, Fig. 1.

Pathway 1 is an SMR-HB national-scale plant to supply an annual demand of 1.6 million tonnes (t) NH_3_ (Tran et al. 2021) up to 4,800 km away. For regional supply to farms up to 800 km away, plants with a capacity of 320,000 t NH_3_ were considered. For these regional-scale plants, two plausible options for this production volume were compared: pathway 2 using conventional SMR-HB plants and pathway 3 using HTP-HB plants. Pathway 4 is for electrolyser-eHB county-scale plants with a capacity of 106,500 t NH_3_ per year, up to 300 km away from farms. And for the local-scale production, the feasible options are electrolyser-eHB plants (pathway 5) and electrolyser-NTP plants (pathway 6) at farmers’ cooperatives with capacity of 10,650 t NH_3_ per year, around 30 km away from farms. Given the high energy demand of the national- and regional-scale plants, fossil NG and electricity from the Australian grid were used in pathways 1, 2, and 3. Despite the potential use of solar photovoltaic electricity or methane from biomass residues in Australia, these energy sources were not considered for the national and regional pathways because they would require large extensions of land (in the case of solar energy) and large quantities of biomass, which are not always available due to its seasonal nature. In the case of the county- and cooperative-scale plants, due to the lower energy demand of these all-electric plants, solar photovoltaic (570kWp open ground installation, multi-Si) systems were considered.

The defined functional unit was 1 tonne of anhydrous NH_3_ produced, stored, and transported to the site of utilisation. The pathways were analysed with the approach of the “avoided burden” or system expansion (ISO 2006; Ayer et al. 2007) because each pathway generates co-products (carbon black (CB) from the HTP process or oxygen (O_2_) from the electrolysis process) and by-products (heat or steam from the HB process).

### Life cycle inventory analysis

Materials, energy, and emissions balances for the NH_3_ production and storage were elaborated based on data from literature, mostly obtained from lab experiments and process simulation software. Then, datasets for materials and energy flows were taken from the available data for Australia in Ecoinvent 3.8 (ETH 2022) cut-off approach. Similarly, to elaborate inventories of the components of transport systems, such as fuel production, vehicle and road manufacturing/construction, maintenance, and end-of-life activities, we adapted the Ecoinvent datasets for road freight transport to our specific study by replacing the average electricity, fuel and biofuel inputs with specific inputs that reflect the Australian mix of sources. In contrast, for the central component of transport systems (i.e., vehicle operation), we used the European air pollutant emission inventory EMEP/EAA guidebook (EEA 2019) to model emissions from fuel, lube oil, and urea consumption, as well as from brake, tyre, and road surface abrasion.

#### Production and storage

Inventories for NH_3_ production in SMR-HB plants in pathways 1 and 2 were elaborated based on the existing model in Aspen software (Anastasopoulou et al. 2020a). For pathway 3, the HTP section consumed 17.3 MWh/t H_2_ and 0.19 m^3^ NG/t H_2_ and produced 3 t CB/t H_2_ (da Costa Labanca 2020). For pathways 4 and 5, we selected proton exchange membrane (PEM) electrolysers because they have a faster response and more flexibility than alkaline electrolysers, being ideal for the use with renewable energy systems (Nel ASA 2020; Ottosson 2021). PEM electrolysis consumed 55.5 MWh/t H_2_ and 14.3 t deionised water/t H_2_, and produced 8 t O_2_/t H_2_ (NREL 2019; Ottosson 2021), with an O_2_ purity of over 99% (Zeng and Zhang 2010), which is suitable for medical applications.

The PSA unit consumed 0.11 MWh/t N_2_ (Morgan et al. 2014). For the NH_3_ synthesis, it basically requires around 0.18 t of H_2_ and 0.84 of N_2_ to produce 1 t of NH_3_. The data for the HB processes was based on Anastasopoulou et al. (2020a, b). The electricity consumption for the NTP reactor was 28.0 MWh/t NH_3_, based on an energy yield of 35.7 g NH_3_/kWh (Kim et al. 2017). The assumed NH_3_ concentration output was 1.0 mol%, which requires a post-separation system with a recycling loop of the unreacted N_2_/H_2,_ which consumes 0.28 MWh/t NH_3_ (Rouwenhorst and Lefferts 2020), plus 1.29 MWh/t NH_3_ for cooling required in the separation/purification process. The final product is stored in pressurized cylinders at 20 °C and 10 bar with a compression cost of 82 kWh/t NH_3,_ for a total of 29.65 MWh/t NH_3_. This storage type was used only for the cooperative-scale plants because pressurized cylinders have maximum capacities of around 270 t (Bartels 2008), therefore, refrigerated tanks at -33 °C and 1 bar were used for the other bigger plants. The annual cost of refrigeration in these tanks is about 64.15 kWh per tonne of NH_3_ capacity (Bartels 2008). We estimated storages capacities of 155,294 t, 31,059 t, 5,168 t, and 514 t for the national, regional, county, and at cooperative plants, considering the storage of 30 days of production for the national and regional plants and 15 days for the county and at cooperative plants (IFDC/UNIDO 1998; Morgan 2013).

#### Transport

The best supply chain scenario for the centralised plant was considered. The initial 4000-km journey from the national-scale plant to the regional storage was carried out by diesel-electric train, and for the rest of the journeys, tanker trucks of different sizes were used. Given the relevance that transport would play in the cost and environmental profile of long-distance distribution of fertilisers, we modelled the vehicle operation emissions using a detailed approach instead of the generic Ecoinvent inventory datasets for road freight transport (Osorio-Tejada et al. 2022d). This specific modelling considered the characteristics of the Australian fleet and roads, such as control emissions standards, truck sizes, load factor, axles number, speed, and road slopes. Inventories were created for each kind of truck with specific Euro emissions standard technology to elaborate single inventories for both rigid and articulated trucks representing the mix of vehicles with different pre-Euro and Euro equivalent control emissions technologies and specific operating conditions in Australia. These detailed inventories are presented in supplementary material SM1.

#### Techno-economic analysis

Capex for the SMR-HB plants, as well as the HB section in pathway 3 were based on the data presented in Anastasopoulou et al. (2020a). Costs for the HTP section were based on data from da Costa Labanca, (2020); for the PEM electrolyser on NREL, (2019); for the NTP reactor on Dobslaw et al. (2017); and for the PSA unit on Kohl and Nielsen, (1997). The equipment for the NH_3_ separation and compression was based on Anastasopoulou et al. (2020a). All these costs were adapted for each plant size using a scaling factor of 0.8 for electrolysers (Demirhan et al. 2019; Squadrito et al. 2021) and 0.6 for the other equipment (Whitesides 2012), then the estimated costs were updated to US$2020 based on the chemical engineering plant cost indexes (CEPCI) (Jenkins 2021).

Regarding Opex, we assumed a cost of $50/MWh for grid electricity and a conservative cost of $40/MWh for onsite photovoltaic electricity generation based on Fasihi et al. (2021), who stated that the cost of fixed tilted solar photovoltaic in Australia was between 20 and 30 €/MWh in 2020, and potentially halving the cost by 2050. Other utilities cost were estimated according to the Ulrich 2006 method (Ulrich and Vasudevan 2006) based on the NG cost during average conditions of 4 $/MMBTU. About catalysts, the NTP and HB reactors consumed Fe surface catalysts with rates of 1.4 kg and 0.056 kg per t of NH_3_, respectively_,_ with an average cost of $15.5/kg (Anastasopoulou et al. 2020a). Labour costs were calculated using a scaling factor of 0.25 (Peters et al. 2003) based on two technicians required for a 340 t NH_3_/year NTP-based plant as reported by Anastasopoulou et al. (2020a). Regarding co-products, the sale price for CB from the HTP section was assumed 1,000 $/t CB (ChemAnalyst 2021). For the price of pure O_2_ from electrolysers, given the high demand for pure O_2_ in hospitals during Covid-19, the analysis of the profitability of electrolyser plants using O_2_ revenues has become relevant. These O_2_ sale credits also require additional investment and energy for the O2 compression and storage system. Recent studies have found profitable electrolyser plants by considering sale prices for compressed high purity O_2_ of 2 €/kg O_2_ (Nicita et al. 2020), 3 €/kg O_2_ (Maggio et al. 2022), or a range from 1 to 7 €/kg O_2_ (Squadrito et al. 2021). However, since the medical grade market is small and limited, and even more in rural areas where NH_3_ plants would be located, the O_2_ production might eventually have to be sold at other markets where not high purity is required, such as wastewater treatment, aquaculture, or industrial processes. The O_2_ sales revenues might be lower, even obtaining minimum revenues of 0.1 AU$/kg O_2_ (Gurieff et al. 2021). Hence, we have assumed a conservative average sale price of 0.55 $/kg O_2_.

For storage, an investment cost for refrigerated storage of about 950 $ per tonne of NH_3_ capacity was based on data from Boom (2020). For the pressurized tanks required in the cooperative-scale plant, the investment cost was about 3,000 $ per tonne of NH_3_ capacity (Leighty 2021; Nayak-Luke et al. 2021). We assumed a 30-year lifespan and a scaling factor of 0.75 based on Morgan (2013). We considered fixed operating costs (e.g., parcel rent, labour costs, etc.), as a fraction of the total capital investment. For refrigerated storage, this fraction was set at 3% along with the cost of refrigeration (64.15 kWh/t NH_3_), while for pressurized storage, the fraction was 2% (Nayak-Luke et al. 2021). After these considerations, we estimated costs of 8.6, 12.7, 9.9, and 19.1 $/t NH_3_ stored for the national, regional, county, and cooperative storage, respectively, at plant gate. That is, the actual storage costs are 50.3, 41.7, 29.0, and 19.1 $/t NH_3_ for the respective pathways, considering the required intermediate storage before the NH_3_ arrives to farm as shown in Fig. 1.

For transportation, we estimated rates of 0.12, 0.21, and 0.30 $/tkm for NH_3_ transport by articulated tanker truck, bobtail 3 axle, and bobtail 2 axle, respectively, based on data from the Western Australia Department of Transport (Australian Government 2021), and incremental costs for the tanker of 80% and maintenance of 13% (Miedvietzky 2008). These figures are in line with the average cost of 0.33 AU$/tkm estimated in 2018 for NH_3_ truck transport (Bruce et al. 2018). In the case of rail transport, the assumed cost was 0.03 $/tkm, based on the average cost of 0.04 AU$/tkm in 2018 for NH_3_ transport in diesel-electric train (Bruce et al. 2018). Transportation is assumed to be carried out by third-party companies, that is, these rates per tkm already include corporate tax and revenue for these companies. The NH_3_ sale prices delivered at farms were calculated based on an expected net profit margin of 5%, after a corporate tax of 30% for large companies and 25% for small and medium companies (Australian Government 2022).

### Life cycle impacts assessment and monetisation methods

Initially, a pre-selection of relevant environmental impact categories in chemical industry was performed according to Morales-Gonzalez et al. (2019) Maranghi and Brondi, (2020), Osorio-Tejada et al. (2022a), namely climate change, acidification, terrestrial ecotoxicity, freshwater eutrophication, freshwater ecotoxicity, photochemical oxidant formation, human toxicity, ozone depletion, particulate matter, and fossil resource scarcity. However, the selection of impact categories and life cycle impacts assessment (LCIA) method was constrained by the selection of monetisation methods. Given the regionalized nature of the economic variables in monetisation approaches, it is explicitly stated in several methods such as Ecovalue08 (Ahlroth and Finnveden 2011), the Environmental Prices Handbook (de Bruyn et al. 2018), MMG (Allacker et al. 2018), and EVR (Vogtländer et al. 2001), that their MVC are only representative for emission sources in Europe, as countries with different technological development levels have varying abatement costs (Arendt et al. 2020). For this reason, choosing a specific geography can limit the implementation of MVC as the results may vary significantly across regions depending on the adopted monetisation approaches (Amadei et al. 2021). To the best of our knowledge, no specific LCA monetisation method has been developed for the analysed region. Hence, to avoid the high uncertainty of the use of any of the available methods, we have defined a range of different MVC for each impact category. This assessment had to be restricted to the six midpoint impact categories identified by Amadei et al. (2021) and the relevant pre-selected categories. Therefore, we utilized the maximum and minimum MVC for five impact categories (i.e., climate change, ozone depletion, particulate matter, photochemical ozone formation, and acidification) presented by de Bruyn et al. (2010), Schneider-Marin and Lang, (2020), Alberici et al. (2014), and de Bruyn et al. (2018), as well as the MVC presented in the recent approach “Monetisation of sustainability impacts of food production and consumption” (Ponsioen et al. 2020). Most of these impact categories are included in ReCiPe 2016 (Huijbregts et al. 2017), one of the most updated and accepted LCIA methods by scientific community due to its representativeness at global scale (Kobayashi et al. 2022). Accordingly, we obtained the LCIA characterization results using SimaPro 9.3 (PRé Consultants 2022), based on the ReCiPe hierarchical (100 years) perspective. For the impact categories of particulate matter and photochemical oxidant formation, ReCiPe 2008 was utilized because most of the available MVC for these categories are presented in the units used in this version of the LCIA method.

### Interpretation

Given that the current maximum energy efficiency of the NTP reactors for NH_3_ synthesis is still low to be commercially feasible, a sensitivity analysis was performed by considering that it is expected that the energy efficiency could increase about 25 times in the near future. These NTP reactors would reduce the energy consumption to 4 GJ/t NH_3_ (Rouwenhorst and Lefferts 2020), i.e., the electricity expenditure would be reduced from 28 to 1.11 MWh/t NH_3_. In this sense, a new plant with this highest NTP energy yield was entitled Pathway 6’. Moreover, the results after internalising the environmental costs into the producers’ NH_3_ sale price are discussed and compared to another way of using the monetized environmental costs in the decision-making of public administrations.

## Results and discussion

**Fig. 2:**
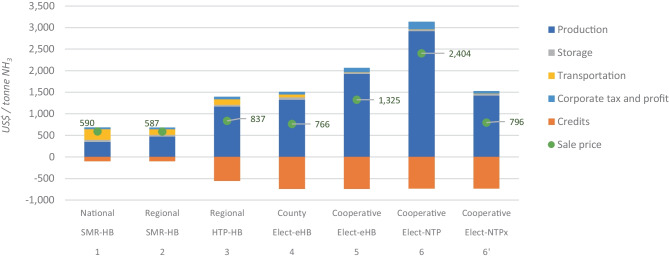
Ammonia supply chain costs and sale price in different pathways. Note: only the most relevant co-product in each pathway is shown as credit, i.e., steam, carbon black, and oxygen for the SMR-HB, HTP-HB, and electrolyser-based plants, respectively. Other less relevant credits, such as heat, CO_2_, hydrogen sulphide, N_2_, H_2_, and NG are already discounted in the production cost

The estimated cost for each delivered tonne of NH_3_ at farms, i.e., the sale prices for the different pathways, are presented in Fig. 2.

**Fig. 3:**
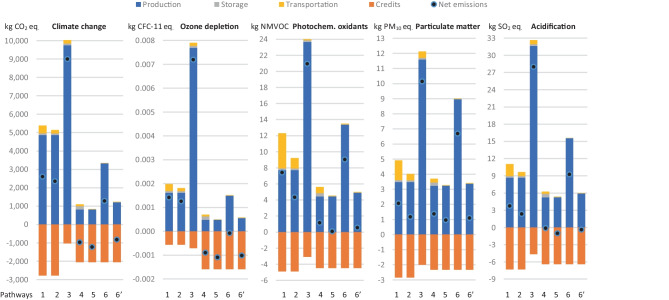
Environmental impacts of ammonia production in different pathways. Note: only the most relevant co-product in each pathway is shown as credit, i.e., steam, carbon black, and oxygen for the SMR-HB, HTP-HB, and electrolyser-based plants, respectively. Other less relevant credits, such as heat, CO_2_, hydrogen sulphide, N_2_, H_2_, and NG are already discounted in the impacts of NH_3_ production

For the conventional NH_3_ pathways, the cost of inland transport can represent up to 41.7% of the NH_3_ wholesale price when. In contrast, transport cost can represent only 1.1% of the NH_3_ wholesale price when it is produced at farm cooperatives. Regarding the contribution of storage in the NH_3_ price, it is only about 8.5% when it is produced in SMR-HB plants at national scale and is slightly reduced in the alternative routes. Among the different alternatives, the conventional pathways 1 and 2 generated the lowest NH_3_ price around 590 $/t NH_3_. Production costs of the conventional plant at regional scale are higher, but these costs are compensated by the lower storage and transportation costs (in grey and yellow in Fig. 2). Despite the lower transport costs and the revenue of credits sales of the alternative pathways, they produce NH_3_ at more than twice the cost of the conventional pathways. The alternative plant with the lowest production costs was the HTP-NG plant of about 1,168 $/t NH_3_, which considering the CB credits, the sale price is 837 $/t NH_3_. However, due to the high quantity of O_2_ credits_,_ the electrolyser-eHB plant at county-scale had the lowest sale price of 766 $/t NH_3_. Yet, given the use of the HB process, this pathway is highly affected when the production scale is reduced, being less competitive in pathway 5. Nevertheless, these alternative pathways presented environmental benefits when supplied by solar photovoltaic energy, as shown in Fig. 3.

It has been found that steam credits in SMR-based plants generate a much higher reduction in environmental impacts than production costs, as shown in the orange bars in Figs. 2 and 3. However, despite the benefits of steam credits, they are not sufficient to compensate for the high environmental impacts of NH_3_ production in SMR-based plants. In fact, the conventional pathways had worse environmental performance than alternative plants, except for the HTP-based plant. SMR-HB plants generated 2.35–2.60 t CO_2_ eq./t NH_3_, while regional plants using HTP technology had the worst performance by generating 9 t CO_2_ eq./t NH_3_ because these plants consume more electricity and NG than SMR-based plants. In these HTP plants, the most relevant source of emissions was the electricity input because it mostly comes from coal and lignite power plants in Australia. For this reason, when solar energy is used, the environmental impacts are highly reduced, especially in the climate change impact category, where electrolyser-based plants have a rate of up to -1.22 t CO_2_ eq./t NH_3_. NTP-based plants can release 1.28 t CO_2_ eq./t NH_3_ and, in the future, these plants could have emissions of about -0,83 t CO_2_ eq./t NH_3._ The inversion of the emissions balance is mainly promoted by the O_2_ sales because the traditional methods for O_2_ production consume a lot of grid electricity, reducing in this way the market for this pollutant traditional-made O_2_.

The simplified supply chain for the decentralised plants also played an important role by reducing the impacts of transport, which accounted for up to 16% of the net carbon footprint and up to 59% of the net photochemical oxidant formation of centralised produced NH_3_. Besides the long distances from the conventional NH_3_ plants to farms, the special characteristics of the load, trucking fleet, roads, and energy sources in Australia contributed to the relevant environmental impacts of NH_3_ distribution. Only for climate change, the CO_2_ eq. emissions per tkm were double when using specific modelling compared to the Ecoinvent dataset. While the generic Ecoinvent dataset shows an emissions rate of 0.14 kg CO_2_ eq./tkm, the modelled articulated and rigid truck emitted 0.17 and 0.30 kg CO_2_ eq./tkm, respectively. The increase in emissions rate was due to higher fuel consumption and also the higher footprint of the production of low-sulphur diesel B5. The production of this fuel with 5% v/v content of vegetable oil methyl ester generates about 17% more carbon emissions than the 100% fossil fuel due to the considerable carbon footprint of palm oil crops in Southeast Asia (Grainger 2013; EFE 2015; Vijay et al. 2016). Diesel-electric trains in Australia also use diesel B5, resulting in a rise in emissions rates from 0.052 to 0.060 kg CO_2_ eq./tkm. Additionally, the high carbon intensity of the Australian electricity also contributes to the high carbon emission rates of transport because it powers vehicle maintenance and road operations.

**Fig. 4:**
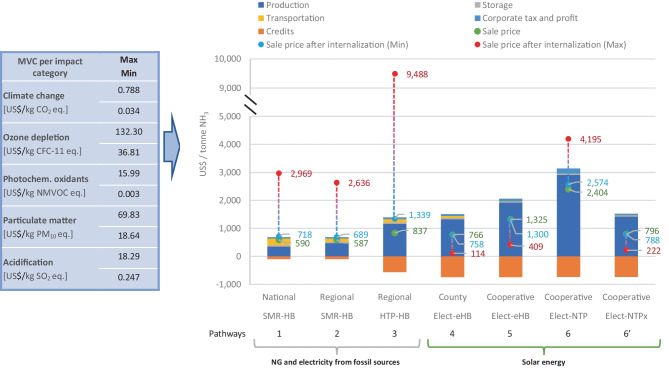
Internalisation of environmental impacts of ammonia production of different pathways in the sale price. Note: only the most relevant co-product in each pathway is shown as credit, i.e., steam, carbon black, and oxygen for the SMR-HB, HTP-HB, and electrolyser-based plants, respectively. Other less relevant credits, such as heat, CO_2_, hydrogen sulphide, N_2_, H_2_, and NG are already discounted in the production cost. Underlying data to create this chart is presented in supplementary material SM2

The obtained environmental results were internalised in the NH_3_ production costs by using MVC, presented in Fig. 4.

Among the MCV per impact category, the highest external costs per kg of emission are for CFC-11 eq. and PM_10_ eq._,_ as shown in the blue table in Fig. 4. The pathways releasing high quantities of these emissions were affected by the internalisation of environmental impacts, such as the HTP-HB regional plant and the electrolyser-NTP local plant, costing in total 9,488 and 4,195 $/t NH_3._ The NTP-based plant, despite being all-electric and supplied by renewable energy, did not obtain the expected improvements because the solar photovoltaic electricity has relevant environmental impacts in its life cycle, mainly related to the manufacturing of solar panels. Particularly, most of the high impacts on photochemical oxidant formation and particulate matter from plants that use solar energy are attributed to the production of high-purity polycrystalline silicon in China. This process requires a substantial amount of electricity, which is primarily generated from hard coal, resulting in the emission of high levels of NOx and particulates.

Nevertheless, despite the range of external cost per kg of CO_2_ eq. is the lowest among the selected emissions, climate change costs are very relevant in the total external costs. For pathways using fossil NG, climate change costs account for over 80% and 60% of the total external costs when the max and the min MVC were considered, respectively. This is because, in these pathways, the net CO_2_ eq emissions are over 2,000 kg/t NH_3_, in comparison to only the PM_10_ eq. and CFC-11 eq emissions which are up to 10 kg/t NH_3_ and 0.007 kg NH_3_. In contrast, for the NTP-based plant (pathway 6), the participation of climate change cost in the total external costs was 56% and 26% when the max and the min MVC were considered, respectively. This is because the other impacts related to solar energy such as particulate matter become relevant, which can share 26% and 73% of the total external costs when the max and min MVC are considered for pathway 6, respectively. These particulate matter emissions become more relevant when using min MVC in all pathways, as opposed to the climate change costs, because the defined min MVC (18.64 $/kg PM_10_ eq.) is only 3.7 times smaller than its max MCV (69.83 $/kg PM_10_ eq.), compared to the range for climate change where the min MVC is 23.2 times smaller than its max MVC. Detailed contributions of environmental impacts in the total external costs can be obtained from tables for internalisation charts in supplementary material SM2.

For the pathways 4, 5, and 6’ where the avoided emissions of the O_2_ sales offset or even inverted the emissions balances of NH_3_ production for climate change, ozone depletion, and acidification impacts, the NH_3_ sale prices after internalisation are below the estimated sale price in Fig. 2. This is because it was assumed that producers would have received a bonus due to emissions reduction. In this sense, the pathways considering the electrolyser-eHB plant at county scale overperformed the novel NTP-based plants, obtaining final sale prices of about 114 $/t NH_3_. Nevertheless, at cooperative scale, the future scenario for NTP-based plants would be more competitive (222 $/t NH_3_) than electrolyser-eHB plants (409 $/t NH_3_) because of the overrun of scaled-down HB plants.

From Fig. 4, it could be concluded that even if bonuses for the environmental benefits of the alternative plants are not considered, and only the taxes due to pollution are charged, the electrolyser-eHB (at county scale) and future NTP-based plants at cooperative scale would be competitive. However, this scenario would hardly occur because imposing such high environmental taxes on traditionally manufactured NH_3_ would make this industry less competitive not only against cleaner local NH_3_ production but also with the international NH_3_ market. Since NH_3_ is a commodity, additional taxes on this product would significantly affect exports, which would not be viewed favourably by the public administration due to the significant loss of income for the country. In this sense, instead of internalising the monetised environmental impacts into each tonne of manufactured NH_3_, another way of using these MVC would be for the estimation of the total environmental costs of the national NH_3_ market. In this way, public administrations could be aware of the amount of money required to mitigate or repair the damages to human health, ecosystems and resources caused by the conventional NH_3_ industry, money that would be saved if cleaner NH_3_ is produced. These estimates for the expected money savings might be useful to take decisions about the budgets that could be invested to promote novel technologies for local NH_3_ production using renewable energies.

**Fig. 5:**
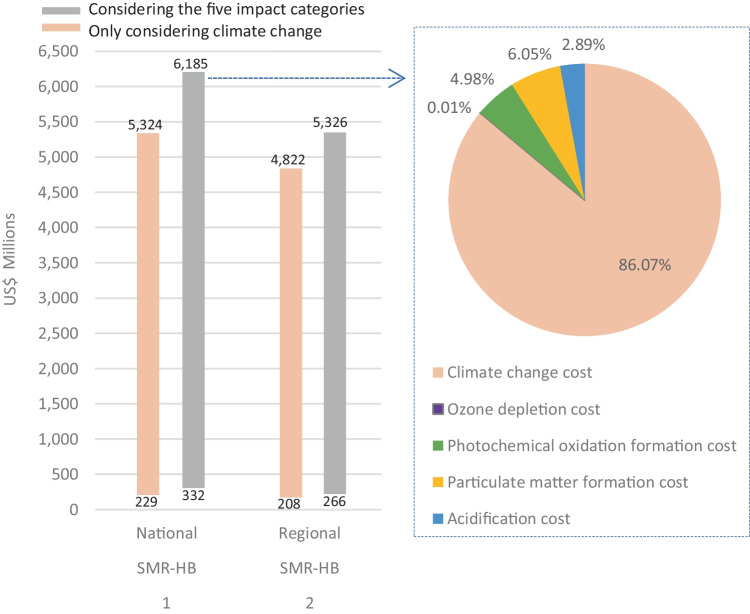
Total external costs for the annual demand of conventionally manufactured NH_3_ in Australia using min and max MVC

For this case study in Australia, the national NH_3_ demand is approximately 2.6 million tonnes per year (Tran et al. 2021). In this sense, if we consider only the external costs related to the CO_2_ emissions of the conventional NH_3_, the total expenditures for public administration in Australia would be between 208 and 5,324 million US$ per year due to the emissions of this industry. In the scenario of considering the external costs of the five impact categories evaluated in this study, the annual expenditures would be between 266 and 6,185 million US$, as shown in Fig. 5.

In Fig. 5, the external costs due to CO_2_ emissions are presented separately given the relevance and focus of the current environmental policy towards the net zero carbon scenarios. Moreover, we can see that when the other four impact categories are considered, the total external costs only increase by 16%. This means that most of these environmental costs in this analysis are related to the carbon emissions, even though each kg of carbon emissions costs less than other emissions such as of CFC-11 eq. or PM_10_ eq. Hence, given the high amount of carbon emitted in the SMR-HB pathway, the climate change costs correspond to 86% of the total external costs of the conventional NH_3_ industry. In this sense, if the max MVC are considered, budgets of around US$5 billion to promote cleaner technologies for NH_3_ synthesis could be considered an investment rather than an expense by public administration in Australia.

## Conclusions

Decentralised and renewable energy-based production of fertilizers has been shown to emit fewer pollutants compared to conventional production pathways. However, despite the environmental benefits, the economic viability of these green fertilizers remains a barrier to the adoption of alternative technologies. The internalisation of environmental impacts of NH_3_ production, as well as the inclusion of impacts of transport, storage, and utilization of by-products, is proposed as a tool to support the transition towards a more sustainable fertiliser supply chain. Internalisation of environmental impacts into monetary cost involves incorporating the external costs or impacts identified in the environmental evaluation into the decision-making process by assigning a monetary value to these impacts. In this way, the full costs of the NH_3_ supply chain are accounted for, including the environmental costs that are often externalised and borne by society. Particularly, the LCA and TEA of novel modular and full electric plants have been performed to determine their competitiveness with conventional SMR-HB plants for a more local NH_3_ production. The 16% of the total 2.60 t CO_2_ eq. emitted per NH_3_ produce at national-scale SMR-HB plants corresponded transport, share that is reduced to 7.5% and 1% when NH_3_ at regional and farm-scale plants, respectively. If other impact categories are analysed, such as net photochemical oxidant formation, transport shares 59% of the impacts of centralised produce NH_3_. The use of alternative technologies fuelled by solar energy and using O_2_ by-product from electrolyser can reduce and even invert the emission balances, generating up to -1.22 t CO_2_ eq./t NH_3_ in electrolyser-eHB plants and -0,83 t CO_2_ eq./t NH_3_ in future NTP-based plants. This reduction is mainly promoted by reducing the market for traditional-made O_2_ using fossil-based electricity mix in Australia. According to the TEA, SMR-HB plants produce the cheapest NH_3_, with sale prices around $590 per ton, while electrolyser-eHB plants have prices ranging between $766 and $1,325 per ton, and NTP-based plants have prices ranging between $796 and $2,404 per ton of NH_3._ After the internalisation of environmental impacts, the electrolyser-eHB plants showed the lowest NH_3_ sale prices than the novel NTP-based plants and the conventional SMR-HB. These later plants would have to sell NH_3_ at prices over 2,636 $/t NH_3,_ while the electrolyser-eHB plant reaches rates of 114 $/t NH_3_. This is due to use the optimized energy usage of the HB processes, which is much higher than the NTP process. However, is important to know that the HB process has reached its efficiency limits, while the NTP process still has room for improvement, as well as its production costs are lower at local-scale plants. In this future scenario of the best energy efficiencies for NTP-based plants, this alternative could also be competitive with rates of 222 $/t NH_3_. In addition, if monetised environmental costs are estimated for a whole industry instead of internalising them into each tonne of NH_3_, it is found that the total external costs of the conventional NH_3_ industry in Australia could amount to about US$5 billion, which can be invested to promote cleaner technologies for local NH_3_ synthesis given the expected savings for public administrations of using these novel technologies.

### Supplementary Information

Below is the link to the electronic supplementary material.

Supplementary file1 (PDF 770 KB)

Supplementary file2 (XLSX 89 KB)

## Data Availability

Supporting data are available as electronic supplementary material. Additional information would be provided upon request.

## Abbreviations

ACC: Annualised capital cost
ACCR: Annual capital charge ratio
B5: Biodiesel 5% v/v
Capex: Capital costs
CB: Carbon black
CEPCI: Chemical engineering plant cost indexes
CFC-11: Trichlorofluoromethane
CO_2_: Carbon dioxide
EEA: European Environment Agency
eHB: Electric Haber–Bosch
EMEP: European Monitoring and Evaluation Programme
EVR: Eco-costs-Value Ratio
HB: Haber-Bosch
HBEFA: Handbook Emission Factors for Road Transport
HTP: High-temperature plasma
H_2_: Hydrogen
LCA: Life cycle assessment
LCIA: Life cycle impacts assessment
MMBTU: Metric million British thermal units
MMG: Milieugerelateerde Materiaalprestatie van Gebouwelementen
MVC: Monetary valuation coefficients
NG: Natural gas
NG: Natural gas
NH_3_: Ammonia
N_2_: Nitrogen
NO_x_: Nitrogen oxides
NMVOC: Non-methane volatile organic compounds
NTP: Non-thermal plasma
Opex: Operating costs
O_2_: Oxygen
PEF: Product environmental footprint
PEM: Proton exchange membrane
PM_10_: Particulate matter with a diameter of 10 microns or less
PSA: Pressure swing adsorption
SMR: Steam methane reforming
TEA: Techno-economic analysis
UCOP: Unitary cost of production

## References

[CR1] Adepia (2022) Parque Industrial. Ante la Cris. Aliment. que se avecina urgen medidas acertadas para enfrentarla

[CR2] Agencia Agraria de Noticias (2022) Perú importa 1.2 millones de toneladas de fertilizantes sintéticos al año. https://agraria.pe/noticias/peru-importa-1-2-millones-de-toneladas-de-fertilizantes-sint-26839. Accessed 23 Jun 2022

[CR3] Agroberichten Buitenland (2022) Impacts of the conflict between Russia and Ukraine on the Andean countries (Part II). https://www.agroberichtenbuitenland.nl/actueel/nieuws/2022/03/31/impacts-of-the-conflict-between-russia-and-ukraine-on-the-andean-countries-part-ii. Accessed 23 Jun 2022

[CR4] Ahlroth S, Finnveden G (2011). Ecovalue08–A new valuation set for environmental systems analysis tools. J Clean Prod.

[CR5] Alberici S, Boeve S, Breevoort P Van et al (2014) Subsidies and costs of EU energy. Final report

[CR6] Allacker K, Debacker W, Delem L et al (2018) Environmental profile of building elements. Mechelen

[CR7] Amadei AM, De Laurentiis V, Sala S (2021) A review of monetary valuation in life cycle assessment: State of the art and future needs. J Clean Prod 329:129668. 10.1016/J.JCLEPRO.2021.129668

[CR8] Anastasopoulou A, Keijzer R, Butala S et al (2020a) Eco-efficiency analysis of plasma-assisted nitrogen fixation. J Phys D Appl Phys 53. 10.1088/1361-6463/ab71a8

[CR9] Anastasopoulou A, Keijzer R, Patil B (2020). Environmental impact assessment of plasma-assisted and conventional ammonia synthesis routes. J Ind Ecol.

[CR10] Arendt R, Bachmann TM, Motoshita M et al (2020) Comparison of Different Monetization Methods in LCA: A Review. Sustainability 12. 10.3390/su122410493

[CR11] Australian Government (2021) Owner-drivers guide and resources. Western Australia Department of transport. In: Own. drivers cost Calc. https://www.transport.wa.gov.au/Freight-Ports/owner-drivers-guide-and-resources.asp. Accessed 5 Jul 2022

[CR12] Australian Government (2022) Australian taxation office. In: Chang. to Co. tax rates. https://www.ato.gov.au/rates/changes-to-company-tax-rates/?page=1#Company_tax_rates. Accessed 23 Jul 2022

[CR13] Ayer NW, Tyedmers PH, Pelletier NL (2007). Co-product allocation in life cycle assessments of seafood production systems: Review of problems and strategies. Int J Life Cycle Assess.

[CR14] Bartels JR (2008) A feasibility study of implementing an Ammonia Economy. Iowa State University

[CR15] Boom S (2020) Comparison of power-to-X-to-power options for energy storage in 2030. TU Delft

[CR16] Bruce S, Temminghoff M, Hayward J (2018). National Hydrogen Roadmap.

[CR17] Canaj K, Mehmeti A, Morrone D et al (2021) Life cycle-based evaluation of environmental impacts and external costs of treated wastewater reuse for irrigation: A case study in southern Italy. J Clean Prod 293:126142. 10.1016/J.JCLEPRO.2021.126142

[CR18] ChemAnalyst (2021) Carbon Black Price Trend and Forecast. In: Mark. Overv. Quart. End. June 2021. https://www.chemanalyst.com/Pricing-data/carbon-black-42. Accessed 10 Sep 2021

[CR19] da Costa Labanca AR (2020). Carbon black and hydrogen production process analysis. Int J Hydrogen Energy.

[CR20] Davidson MD, Boon BH, Van Swigchem J (2005). Monetary valuation of emissions in implementing environmental policy: The reduction cost approach based upon policy targets. J Ind Ecol.

[CR21] de Bruyn S, Bijleveld M, de Graaff L et al (2018) Environmental Prices Handbook. EU28 version. CE Delft, Delft

[CR22] de Bruyn S, Korteland M, Davidson M, Bles M (2010) Shadow Prices Handbook. Valuation and weighting of emissions and environmental impacts. Delft

[CR23] Demirhan CD, Tso WW, Powell JB, Pistikopoulos EN (2019) Sustainable ammonia production through process synthesis and global optimization. AIChE J 65. 10.1002/aic.16498

[CR24] Dobslaw D, Schulz A, Helbich S (2017). VOC removal and odor abatement by a low-cost plasma enhanced biotrickling filter process. J Environ Chem Eng.

[CR25] Durão V, Silvestre JD, Mateus R, De Brito J (2019) Economic valuation of life cycle environmental impacts of construction products - A critical analysis. IOP Conf Ser Earth Environ Sci 323:012147. 10.1088/1755-1315/323/1/012147

[CR26] Durisin M (2021) Bloomberg. In: Fertil. Spike Hits Eur. Farmers Hear. Plant. https://www.bloomberg.com/news/articles/2021-10-13/fertilizer-spike-hits-european-farmers-during-heart-of-planting. Accessed 15 Oct 2021

[CR27] EEA (2019) EMEP/EEA air pollutant emission inventory guidebook: Technical guidance to prepare national emission inventories. Luxembourg

[CR28] EFE (2015) 20 minutos. Reemplazar bosque Trop. por aceite palma, cacao o caucho aumenta las emisiones CO2

[CR29] ETH (2022) Ecoinvent LCA database. In: Ecoinvent v3.8. www.ecoinvent.org. Accessed 22 May 2021

[CR30] European Commission (2018) PEFCR Guidance document - Guidance for the development of Product Environmental Footprint Category Rules (PEFCRs), version 6.3

[CR31] European Commission, Directorate-General for Energy, Smith M et al (2020) External costs : energy costs, taxes and the impact of government interventions on investments : final report. Publications Office, Rotterdam

[CR32] European Commission, Directorate-General for Mobility and Transport, Essen H et al (2019) Handbook on the external costs of transport : version 2019. Publications Office

[CR33] Fasihi M, Weiss R, Savolainen J, Breyer C (2021) Global potential of green ammonia based on hybrid PV-wind power plants. Appl Energy 294:116170. 10.1016/j.apenergy.2020.116170

[CR34] Fertilizers Australia (2019) Australian Fertilizer Market. https://fertilizer.org.au/fertiliser-industry/australian-fertilizer-market. Accessed 29 May 2023

[CR35] Fúnez-Guerra C, Reyes-Bozo L, Vyhmeister E (2020). Technical-economic analysis for a green ammonia production plant in Chile and its subsequent transport to Japan. Renew Energy.

[CR36] Goedkoop M, Heijungs R, Huijbregts M et al (2009) ReCiPe 2008. Potentials 1–44. 10.1029/2003JD004283

[CR37] Government of Peru (2022) Panorama nacional e internacional del mercado de fertilizantes inorgánicos. https://cdn.www.gob.pe/uploads/document/file/2962887/Mercadodefertilizantesinorgánicos.pdf. Accessed 23 Jun 2022

[CR38] Grainger M (2013) One World News. IPCC underestimating palm oil pollution, say Campaign.

[CR39] Gurieff N, Moghtaderi B, Daiyan R (2021) Gas Transition: Renewable Hydrogen’s Future in Eastern Australia’s Energy Networks. In: Advances in Energy Research, 3rd edn.

[CR40] Huijbregts MAJ, Steinmann ZJN, Elshout PMF et al (2017) ReCiPe 2016 v1.1. A harmonized life cycle impact assessment method at midpoint and endpoint level. Report I: Characterization. Bilthoven

[CR41] IEA (2021) Ammonia Technology Roadmap. Paris

[CR42] IFDC, UNIDO (1998) Fertilizer Manual, 3rd edn. Kluwer Academic Publisher, Norwell, MA

[CR43] IFDC (2019) Structure des Coûts Logistiques et des Procédures d’Importation des Engrais sur 4 Corridors en Afrique de

[CR44] Indexbox (2023) World - Anhydrous Ammonia - Market Analysis, Forecast, Size, Trends and Insights. Luxembourg

[CR45] ISO (2006) ISO 14040:2006. Environmental management — Life cycle assessment — Principles and framework, Second edi. ISO, Geneva

[CR46] Jenkins S (2021) CE Plant Cost Index (CEPCI) update. https://www.chemengonline.com/2021-cepci-updates-june-prelim-and-may-final. Accessed 10 Sep 2021

[CR47] Kim HH, Teramoto Y, Ogata A et al (2017) Atmospheric-pressure nonthermal plasma synthesis of ammonia over ruthenium catalysts. Plasma Process Polym 14. 10.1002/ppap.201600157

[CR48] Kobayashi Y, Kärkkäinen E, Häkkinen ST et al (2022) Life cycle assessment of plant cell cultures. Sci Total Environ 808:151990. 10.1016/j.scitotenv.2021.151990 10.1016/j.scitotenv.2021.151990 34843779

[CR49] Kohl AL, Nielsen RB (1997) Membrane Permeation Processes. In: Gas Purification. Gulf Professional Publishing, pp 1238–1295

[CR50] Leighty B (2021) Energy Storage with Anhydrous Ammonia: Comparison with other Energy Storage. In: Ammonia: The Key to US Energy Independence. Minneapolis, p 74

[CR51] Long NVD, Kim GS, Tran NN (2021). Biogas upgrading using ionic liquid [Bmim][PF6] followed by thermal-plasma-assisted renewable hydrogen and solid carbon production. Int J Hydrogen Energy.

[CR52] Maggio G, Squadrito G, Nicita A (2022) Hydrogen and medical oxygen by renewable energy based electrolysis: A green and economically viable route. Appl Energy 306:117993. 10.1016/J.APENERGY.2021.117993

[CR53] Maranghi S, Brondi C (2020). Life Cycle Assessment in the Chemical Product Chain.

[CR54] Miedvietzky E (2008) Alternativas para el abastecimiento de GLP al NEA. Instituto Tecnologico de Buenos Aires

[CR55] Morales-Gonzalez OM, Escribà-Gelonch M, Hessel V (2019). Life cycle assessment of vitamin D3 synthesis: from batch to photo-high p, T. Int J Life Cycle Assess.

[CR56] Morgan E, Manwell J, McGowan J (2014). Wind-powered ammonia fuel production for remote islands: A case study. Renew Energy.

[CR57] Morgan ER (2013) Techno-economic feasibility study of ammonia plants powered by offshore wind

[CR58] Muelaner J (2020) Engineering.com. In: Using Ammon. to Store Transp. Renew. Energy. https://www.engineering.com/story/using-ammonia-to-store-and-transport-renewable-energy. Accessed 12 Oct 2021

[CR59] Nayak-Luke RM, Forbes C, Cesaro Z et al (2021) Techno-Economic Aspects of Production, Storage and Distribution of Ammonia. In: Techno-Economic Challenges of Green Ammonia as an Energy Vector. Academic Press, pp 191–207

[CR60] Nel ASA (2020) Nel Hydrogen. In: Water electrolysers / Hydrog. Gener. https://nelhydrogen.com/water-electrolysers-hydrogen-generators/. Accessed 2 Jul 2022

[CR61] Nicita A, Maggio G, Andaloro APF, Squadrito G (2020). Green hydrogen as feedstock: Financial analysis of a photovoltaic-powered electrolysis plant. Int J Hydrogen Energy.

[CR62] NREL (2019) H2A: Hydrogen Analysis Production Models. Hydrogen and Fuel Cells. NREL. In: Curr Cent Hydrog Prod from Polym. Electrolyte Membr. Electrolysis version 3.2018. https://www.nrel.gov/hydrogen/h2a-production-models.html. Accessed 10 Sep 2021

[CR63] Osorio-Tejada J, Ferlin F, Vaccaro L, Hessel V (2022). Life cycle assessment of multistep benzoxazole synthesis: from batch to waste-minimised continuous flow systems. Green Chem.

[CR64] Osorio-Tejada J, Tran NN, Hessel V (2022b) Techno-environmental assessment of small-scale Haber-Bosch and plasma-assisted ammonia supply chains. Sci Total Environ 826:154162. 10.1016/j.scitotenv.2022.154162 10.1016/j.scitotenv.2022.154162 35240177

[CR65] Osorio-Tejada J, van’t Veer K, Long NVD et al (2022c) Sustainability analysis of methane-to-hydrogen-to-ammonia conversion by integration of high-temperature plasma and non-thermal plasma processes. Energy Convers Manag 269:116095. 10.1016/J.ENCONMAN.2022.116095

[CR66] Osorio-Tejada JL, Llera-Sastresa E, Scarpellini S (2022). Environmental assessment of road freight transport services beyond the tank-to-wheels analysis based on LCA. Environ Dev Sustain.

[CR67] Ottosson A (2021) Integration of Hydrogen Production via Water Electrolysis at a CHP Plant A feasibility study. Luleå University of Technology

[CR68] Parkinson B, Tabatabaei M, Upham DC (2018). Hydrogen production using methane : Techno- economics of decarbonizing fuels and chemicals. Int J Hydrogen Energy.

[CR69] Peters MS, Timmerhaus KD, West RE (2003) Plant Design and Economics for Chemical Engineers 5th Edition, 5th edn. New York

[CR70] Pizzol M, Weidema B, Brandão M, Osset P (2015). Monetary valuation in Life Cycle Assessment: a review. J Clean Prod.

[CR71] Ponsioen T, Nuhoff-isakhanyan G, Vellinga T (2020). Monetisation of sustainability impacts of food production and consumption.

[CR72] PRé Consultants (2022) SimaPro v9. In: About SimaPro. www.pre.nl/content/simapro-lca-software. Accessed 16 Jan 2022

[CR73] Roberts S, Vilakazi T (2014) Regulation and rivalry in transport and fertilizer supply in Malawi, Tanzania and Zambia

[CR74] Rouwenhorst KHR, Lefferts L (2020) Feasibility Study of Plasma-Catalytic Ammonia Synthesis for Energy Storage Applications. Catalysts 10. 10.3390/catal10090999

[CR75] Sarafraz MM, Tran NN, Nguyen H (2021). Tri-fold process integration leveraging high- and low-temperature plasmas: From biomass to fertilizers with local energy and for local use. J Adv Manuf Process.

[CR76] Schneider-Marin P, Lang W (2020). Environmental costs of buildings: monetary valuation of ecological indicators for the building industry. Int J Life Cycle Assess.

[CR77] Snoeckx R, Bogaerts A (2017). Plasma technology-a novel solution for CO2 conversion?. Chem Soc Rev.

[CR78] Squadrito G, Nicita A, Maggio G (2021). A size-dependent financial evaluation of green hydrogen-oxygen co-production. Renew Energy.

[CR79] Takele TB, Tolcha TD (2021). Optimal transit corridors for Ethiopia. J Transp Supply Chain Manag.

[CR80] The Royal Society (2020) Ammonia: zero-carbon fertiliser, fuel and energy store. London

[CR81] Thi TLN, Laratte B, Guillaume B, Hua A (2016). Quantifying environmental externalities with a view to internalizing them in the price of products, using different monetization models. Resour Conserv Recycl.

[CR82] Towler G, Sinnott R (2013) Economic Evaluation of Projects. In: Butterworth-Heinemann (ed) Chemical Engineering Design: Principles, Practice and Economics of Plant and Process Design, Second. Elsevier Ltd, Oxford, pp 389–429

[CR83] Tran N, Osorio-Tejada J, Asrami MR (2021). Economic Optimization of Local Australian Ammonia Production Using Plasma Technologies with Green/Turquoise Hydrogen. ACS Sustain Chem Eng.

[CR84] Ulrich GD, Vasudevan PT (2006) How to estimate utility costs. Chem. Eng. 113

[CR85] Vijay V, Pimm SL, Jenkins CN, Smith SJ (2016) The Impacts of Oil Palm on Recent Deforestation and Biodiversity Loss. PLoS One 11:e0159668. 10.1371/journal.pone.0159668 10.1371/journal.pone.0159668 PMC4963098 27462984

[CR86] Vilakazi TS (2018). The causes of high intra-regional road freight rates for food and commodities in Southern Africa. Dev South Afr.

[CR87] Vogtländer JG, Brezet HC, Hendriks CF (2001). The virtual eco-costs ‘99 A single LCA-based indicator for sustainability and the eco-costs-value ratio (EVR) model for economic allocation. Int J Life Cycle Assess.

[CR88] Whitesides RW (2012) Process Equipment Cost Estimating by Ratio and Proportion. Course notes, PDH Course G 8

[CR89] Zeng K, Zhang D (2010). Recent progress in alkaline water electrolysis for hydrogen production and applications. Prog Energy Combust Sci.

