# Supplementary material for: Internalisation of environmental costs of decentralised nitrogen fertilisers production
Source: Int J Life Cycle Assess. 2023 Jun 9;28(11):1590–603. doi: 10.1007/s11367-023-02187-5 (PMC10251320; doi:10.1007/s11367-023-02187-5)
Supplement: Supplementary file 1 — Supplementary file1 (PDF 770 KB) [file 11367_2023_2187_MOESM1_ESM.pdf]

SUPPLEMENTARY MATERIAL SM1-

LIFE CYCLE INVENTORY (LCI) DATASETS FOR:

Internalisation of environmental costs of decentralised nitrogen fertilisers production

Jose Osorio-Tejada[1,3*], Evgeny Rebrov[1], Volker Hessel[1,2]

[1]School of Engineering, University of Warwick, Coventry CV4 7AL, UK
[2]School of Chemical Engineering, University of Adelaide, Adelaide SA 5005, Australia
[3]Faculty of Environmental Sciences, Universidad Tecnológica de Pereira, Pereira, Colombia

**Corresponding author: Jose Luis Osorio-Tejada. Tel.: +44-7727959525.*
E-mail address: jose.osorio-tejada@warwick.ac.uk

CONTENTS

Table S1. LCI for Pathway 1
Table S2. LCI for Pathway 2
Table S3. LCI for Pathway 3
Table S4. LCI for Pathway 4
Table S5. LCI for Pathway 5
Table S6. LCI for Pathway 6
Table S7. LCI for Pathway 6’
Table S8. LCI for Hydrogen and Nitrogen production {AU}|Conventional SMR for HB process (Pathways 1&2)
Table S9. LCI for Hydrogen and Nitrogen production {AU}|HTP for HB process (Pathway 3)
Table S10. LCI for Hydrogen and Nitrogen production {AU}|Electrolysis and PSA for eHB process (Pathways 4&5)
Table S11. LCI for Hydrogen and Nitrogen production {AU}|Electrolysis and PSA for NTP process (Pathways 6&6’)
Table S12. Allocation values for NH_3 storage tanks
Table S13. LCI for Truck transport of liquid ammonia in 43.5-ton articulated truck (Australian average)
Table S14. LCI for Truck transport of liquid ammonia in 43.5-ton articulated truck {AU} (PRE-EURO to EURO V).
Table S15. LCI for Transport (only traffic) in 43.5-ton articulated truck {AU} (PRE-EURO to EURO V)
Table S16. LCI for Diesel combustion emissions per tkm 43.5-ton articulated truck {AU} (PRE-EURO to EURO V)
Table S17. LCI for Engine oil consumption emissions per tkm 43.5-ton articulated truck {AU}
Table S18. LCI for Tyre abrasion emissions per tkm 43.5-ton articulated truck {AU}
Table S19. LCI for Brake abrasion emissions per tkm 43.5-ton articulated truck {AU}
Table S20. LCI for Road surface abrasion emissions per tkm 43.5-ton articulated truck {AU}
Table S21. LCI for Urea consumption emissions per tkm 43.5-ton articulated truck {AU} (ONLY FOR EURO V)
Table S22. LCI for Truck transport of liquid ammonia in 15-ton rigid truck (Australian average)
Table S23. LCI for Truck transport of liquid ammonia in 15-ton rigid truck {AU} (PRE-EURO to EURO V)
Table S24. LCI for Transport (only traffic) in 15-ton rigid truck {AU} (PRE-EURO to EURO V)
Table S25. LCI for Diesel combustion emissions per tkm 15-ton rigid truck {AU} (PRE-EURO to EURO V)
Table S26. LCI for Engine oil consumption emissions per tkm 15-ton rigid truck {AU}
Table S27. LCI for Tyre abrasion emissions per tkm 15-ton rigid truck {AU}
Table S28. LCI for Brake abrasion emissions per tkm 15-ton rigid truck {AU}
Table S29. LCI for Road surface abrasion emissions per tkm 15-ton rigid truck {AU}
Table S30. LCI for Urea consumption emissions per tkm 15-ton rigid truck {AU} (ONLY FOR EURO V)

Life cycle inventories for ammonia production and storage

In Tables S1-S7, the life cycle inventories (LCI) for the NH_3 synthesis, storage, and transport in each pathway are presented. In each of these datasets, there is an item named "Hydrogen and Nitrogen production {AU}…" with the amount equal to 1 unit (p). This item corresponds to the process to produce nitrogen using pressure swing adsorption and hydrogen using the respective technology for each pathway (i.e., steam methane reforming, high-temperature plasma, or electrolysis). These LCI are presented in separate datasets in Tables S8-S11 in order to isolate the section of the production of feedstocks supply and the section of NH_3 synthesis and storage. The chemicals plant infrastructure is included in the feedstocks section.

Table S1. LCI for Pathway 1

Products	Amount	unit
Pathway 1 - Ammonia, liquid Conventional Haber-Bosch reactor (TRAIN + TRUCK specific)	1	t
**Avoided products**		
Steam, in chemical industry {RoW}| production	*9.39E+02*	*kg*
Natural gas, high pressure {RoW} production	*9.48E+01*	*m^3*
Hydrogen, liquid {AU}| hydrogen cracking, APME, production	*7.39E+00*	*kg*
**Materials/fuels**		
Hydrogen and Nitrogen production {AU}| Conventional SMR for HB process	*1*	*p*
Water, deionised {AU} | market for water, deionised	*9.39E+02*	*kg*
Liquid storage tank, chemicals, organics {AU}| production	*9.96E-07*	*p*
Transport, freight train {AU}| diesel B5	*4.00E+03*	*tkm*
Truck transport liquid ammonia in 43.5-ton articulated truck {AU} AUSTRALIAN AVERAGE	*5.00E+02*	*tkm*
Truck transport liquid ammonia in 15-ton rigid truck {AU} AUSTRALIAN AVERAGE	*3.00E+02*	*tkm*
**Electricity/heat**		
Electricity, medium voltage {AU}| market for (production)	*3.96E+02*	*kWh*
Electricity, medium voltage {AU}| market for (storage)	*1.55E+01*	*kWh*
Cooling energy {RoW}| from natural gas, at cogen unit with absorption chiller 100kW	*3.89E+03*	*MJ*
Heat, district or industrial, natural gas {RoW}| heat production, natural gas, at industrial furnace low-NOx >100kW	*5.10E+01*	*MJ*
**Emissions to air**		
**Emissions to water**		
**Emissions to soil**		
**Waste to treatment**		

Table S2. LCI for Pathway 2

Products	Amount	unit
Pathway 2 - Ammonia, liquid Conventional Haber-Bosch reactor (TRUCK specific)	1	t
**Avoided products**		
Steam, in chemical industry {RoW}| production	*9.39E+02*	*kg*
Natural gas, high pressure {RoW} production	*9.48E+01*	*m^3*
Hydrogen, liquid {AU}| hydrogen cracking, APME, production	*7.39E+00*	*kg*
**Materials/fuels**		
Hydrogen and Nitrogen production {AU}| Conventional SMR for HB process	*1*	*p*
Water, deionised {AU} | market for water, deionised	*9.39E+02*	*kg*
Liquid storage tank, chemicals, organics {AU}| production	*6.64E-07*	*p*
Truck transport liquid ammonia in 43.5-ton articulated truck {AU} AUSTRALIAN AVERAGE	*5.00E+02*	*tkm*
Truck transport liquid ammonia in 15-ton rigid truck {AU} AUSTRALIAN AVERAGE	*3.00E+02*	*tkm*
**Electricity/heat**		
Electricity, medium voltage {AU}| market for (production)	*3.96E+02*	*kWh*
Electricity, medium voltage {AU}| market for (storage)	*9.32E+00*	*kWh*
Cooling energy {RoW}| from natural gas, at cogen unit with absorption chiller 100kW	*3.89E+03*	*MJ*
Heat, district or industrial, natural gas {RoW}| heat production, natural gas, at industrial furnace low-NOx >100kW	*5.10E+01*	*MJ*
**Emissions to air**		
**Emissions to water**		
**Emissions to soil**		
**Waste to treatment**		

Table S3. LCI for Pathway 3

Products	Amount	unit
Pathway 3 - HTP - HB reactor	1	t
**Avoided products**		
Nitrogen, liquid {AU}| air separation, cryogenic, production	*1.80E+01*	kg
Hydrogen, liquid {AU}| hydrogen cracking, APME, production	*5.00E+00*	kg
Steam, in chemical industry {RoW}| production	*1.08E+03*	kg
**Materials/fuels**		
Hydrogen and Nitrogen production {AU}| HTP for HB process	*1*	p
Water, deionised {AU} | market for water, deionised	*1.09E+03*	kg
Liquid storage tank, chemicals, organics {AU}| production	*6.64E-07*	p
Truck transport liquid ammonia in 43.5-ton articulated truck {AU} AUSTRALIAN AVERAGE	*5.00E+02*	tkm
Truck transport liquid ammonia in 15-ton rigid truck {AU} AUSTRALIAN AVERAGE	*3.00E+02*	tkm
**Electricity/heat**		
Electricity, medium voltage {AU}| market for (production)	*5.42E+03*	*kWh*
Electricity, medium voltage {AU}| market for (storage)	*9.32E+00*	*kWh*
Cooling energy {AU}| with electric chiller	*1.67E+04*	*MJ*
Heat, district or industrial, natural gas {AU}| heat production, natural gas, at industrial furnace low-NOx >100kW	*1.70E+01*	*MJ*
**Emissions to air**		
**Emissions to water**		
**Emissions to soil**		
**Waste to treatment**		

Table S4. LCI for Pathway 4

Products	Amount	unit
Pathway 4 - Electrolysis - eHB reactor	1	t
**Avoided products**		
Nitrogen, liquid {AU}| air separation, cryogenic, production	*1.80E+01*	kg
Hydrogen, liquid {AU}| hydrogen cracking, APME, production	*5.00E+00*	kg
Steam, in chemical industry {RoW}| production	*1.08E+03*	kg
**Materials/fuels**		
Hydrogen and Nitrogen production {AU}| Electrolysis and PSA for eHB process	*1*	p
Water, deionised {AU} | market for water, deionised	*1.09E+03*	kg
Liquid storage tank, chemicals, organics {AU}| production	*3.32E-07*	p
Truck transport liquid ammonia in 15-ton rigid truck {AU} AUSTRALIAN AVERAGE	*3.00E+02*	tkm
**Electricity/heat**		
Electricity, low voltage {AU}| electricity production, photovoltaic, 570kWp open ground, multi-Si (production)	*6.19E+03*	*kWh*
Electricity, low voltage {AU}| electricity production, photovoltaic, 570kWp open ground, multi-Si (storage)	*3.11E+00*	*kWh*
Cooling energy {AU}| with electric chiller, SOLAR energy	*1.54E+04*	*MJ*
**Emissions to air**		
**Emissions to water**		
**Emissions to soil**		
**Waste to treatment**		

Table S5. LCI for Pathway 5

Products	Amount	unit
Pathway 5 - Electrolysis - eHB reactor	1	t
**Avoided products**		
Nitrogen, liquid {AU}| air separation, cryogenic, production	*1.80E+01*	kg
Hydrogen, liquid {AU}| hydrogen cracking, APME, production	*5.00E+00*	kg
Steam, in chemical industry {RoW}| production	*1.08E+03*	kg
**Materials/fuels**		
Hydrogen and Nitrogen production {AU}| Electrolysis and PSA for eHB process	*1*	p
Water, deionised {AU} | market for water, deionised	*1.09E+03*	kg
Liquid storage tank, chemicals, organics {AU}| production	*6.64E-07*	p
Truck transport liquid ammonia in 15-ton rigid truck {AU} AUSTRALIAN AVERAGE	*3.00E+02*	tkm
**Electricity/heat**		
Electricity, low voltage {AU}| electricity production, photovoltaic, 570kWp open ground, multi-Si (production)	*6.19E+03*	*kWh*
Cooling energy {AU}| with electric chiller, SOLAR energy	*1.54E+04*	*MJ*
**Emissions to air**		
**Emissions to water**		
**Emissions to soil**		
**Waste to treatment**		

Table S6. LCI for Pathway 6

Products	Amount	unit
Pathway 6 - Electrolysis - NTP reactor	1	t
**Avoided products**		
**Materials/fuels**		
Hydrogen and Nitrogen production {AU}| Electrolysis and PSA for NTP process	*1*	p
Liquid storage tank, chemicals, organics {AU}| production	3.32E-07	p
Truck transport liquid ammonia in 15-ton rigid truck {AU} AUSTRALIAN AVERAGE	*3.00E+01*	tkm
**Electricity/heat**		
Electricity, low voltage {AU}| electricity production, photovoltaic, 570kWp open ground, multi-Si (production)	*2.97E+04*	*kWh*
**Emissions to air**		
**Emissions to water**		
**Emissions to soil**		
**Waste to treatment**		

Table S7. LCI for Pathway 6’

Products	Amount	unit
Pathway 6’ - Electrolysis - NTPx reactor	1	t
**Avoided products**		
**Materials/fuels**		
Hydrogen and Nitrogen production {AU}| Electrolysis and PSA for NTP process	*1*	p
Liquid storage tank, chemicals, organics {AU}| production	3.32E-07	p
Truck transport liquid ammonia in 15-ton rigid truck {AU} AUSTRALIAN AVERAGE	*3.00E+01*	tkm
**Electricity/heat**		
Electricity, low voltage {AU}| electricity production, photovoltaic, 570kWp open ground, multi-Si (production)	*2.68E+03*	*kWh*
**Emissions to air**		
**Emissions to water**		
**Emissions to soil**		
**Waste to treatment**		

Table S8. LCI for Hydrogen and Nitrogen production {AU}| Conventional SMR for HB process (Pathways 1 and 2)

Products	Amount	unit
Hydrogen and Nitrogen production {AU}| Conventional SMR for HB process	1	t
**Avoided products**		
Hydrogen sulfide {AU}| production	*7.00E-03*	kg
Steam, in chemical industry {RoW}| production	*7.32E+03*	kg
Carbon dioxide, liquid {AU}| production	*1.00E-01*	kg
**Materials/fuels**		
Natural gas, high pressure {GLO}| market group for	*6.27E+02*	*m³*
Steam, in chemical industry {RoW}| production	*1.83E+03*	*kg*
Water, deionised {AU}| market for water, deionised	*7.32E+03*	*kg*
Chemical factory, organics {GLO}| market for	*6.66E-07*	*p*
**Electricity/heat**		
Heat, district or industrial, natural gas {AU}| heat production, natural gas, at industrial furnace low-NOx >100kW	*1.69E+04*	*MJ*
Electricity, medium voltage {AU}| market for	*1.15E+02*	*kWh*
Cooling energy {AU}| from natural gas (RoW), at cogen unit with absorption chiller 100kW	*1.03E+04*	*MJ*
**Emissions to air**		
**Emissions to water**		
**Emissions to soil**		
**Waste to treatment**		

Table S9. LCI for Hydrogen and Nitrogen production {AU}| HTP for HB process (Pathway 3)

Products	Amount	unit
Hydrogen and Nitrogen production {AU}| HTP for HB process	1	t
**Avoided products**		
Carbon black {AU}| production	*5.58E+02*	*kg*
**Materials/fuels**		
Chemical factory, organics {GLO}| market for	*6.67E-07*	*p*
**Electricity/heat**		
Electricity, medium voltage {AU}| market for	*3.30E+03*	*kWh*
Natural gas, high pressure {GLO}| market group for	*1.01E+03*	*m³*
Cooling energy {AU}| from natural gas, at cogen unit with absorption chiller 100kW	*7.53E+02*	*MJ*
**Emissions to air**		
**Emissions to water**		
**Emissions to soil**		
**Waste to treatment**		

Table S10. LCI for Hydrogen and Nitrogen production {AU}| Electrolysis and PSA for eHB process (Pathways 4 and 5)

Products	Amount	unit
Hydrogen and Nitrogen production {AU}| Electrolysis and PSA for eHB process	1	t
**Avoided products**		
Oxygen, liquid {AU}| air separation, cryogenic	*1.46E+03*	*kg*
**Materials/fuels**		
Chemical factory, organics {GLO}| market for	*6.67E-07*	*p*
Water, deionised {AU}| market for water, deionised	*2.66E+03*	*kg*
**Electricity/heat**		
Electricity, low voltage {AU}| electricity production, photovoltaic, 570kWp open ground installation, multi-Si	*1.06E+04*	*kWh*
Cooling energy {AU}| with electric chiller | Cut-off, U SOLAR	*7.60E+02*	*MJ*
**Emissions to air**		
**Emissions to water**		
**Emissions to soil**		
**Waste to treatment**		

Table S11. LCI for Hydrogen and Nitrogen production {AU}| Electrolysis and PSA for NTP process (Pathways 6 and 6')

Products	Amount	unit
Hydrogen and Nitrogen production {AU}| Electrolysis and PSA for NTP process	1	t
**Avoided products**		
Oxygen, liquid {AU}| air separation, cryogenic	*1.46E+03*	*kg*
**Materials/fuels**		
Chemical factory, organics {GLO}| market for	*6.67E-07*	*p*
Water, deionised {AU}| market for water, deionised	*2.59E+03*	*kg*
**Electricity/heat**		
Electricity, low voltage {AU}| electricity production, photovoltaic, 570kWp open ground installation, multi-Si	*1.03E+04*	*kWh*
**Emissions to air**		
**Emissions to water**		
**Emissions to soil**		
**Waste to treatment**		

Table S12. Allocation values for NH_3 storage tanks

Items	Storage size			
	National	Regional	County	Cooperative
Plant production (t/day)	4705.9	941.2	313.2	31.2
Storage (days)	30	30	15	15
Operating time (days/year)	340	340	340	340
Freeboard (%)	10%	10%	10%	10%
Lifespan (years)	30	30	30	30
Energy for refrigeration(kWh/t.year^{-1})	64	64	64	0
NH_3 density (t/m^3)	0.609	0.609	0.609	0.609
Storage capacity of Ecoinvent dataset (m^3)	16,000	16,000	16,000	16,000
Required storage capacity (m^3)	254,999	51,000	8,487	845
Required storage capacity (t)	155,294	31,059	5,168	514
Total NH_3 stored in a year (t/year)	1,600,000	320,000	106,500	10,600
Total NH_3 stored in the useful like (t)	48,000,000	9,600,000	3,195,000	318,000
Allocation per NH_3 tonne of a tank (16,000 m^3)	2.08E-08	1.04E-07	3.13E-07	3.14E-06
Number of tanks of 16,000 m^3 required	15.937	3.187	0.530	0.053
Allocation per NH_3 tonne for the required tank	**3.32E-07**	**3.32E-07**	**1.66E-07**	**1.66E-07**
Energy consumption per NH_3 tonne (kWh/t)	**6.2**	**6.2**	**3.1**	**0.00**
**Cumulative allocation values**	**9.96E-07**	**6.64E-07**	**3.32E-07**	**1.66E-07**
**Cumulative energy consumption values (kWh/t)**	**15.53**	**9.32**	**3.11**	**0.00**

Life cycle inventories for ammonia transport

The specific modelling of the transport process considered the characteristics of the Australian fleet and roads, such as control emissions standards, truck sizes, load factor, axles number, speed, and road slopes. Inventories were created for each kind of truck with specific Euro emissions standard technology to elaborate single inventories for both rigid and articulated trucks representing the mix of vehicles with different pre-Euro and Euro equivalent control emissions technologies and specific operating conditions in Australia. There are several reasons why this approach was preferable: *(i)* while Ecoinvent datasets for Europe and rest-of-the-world include vehicles only with Euro III, IV, V, and VI emissions standards, and specific datasets for vehicles with Euro I and II standards in South Africa, the majority of Australian fleet have older technologies than Euro III, with 35% of rigid trucks having pre-Euro standards, SAFC, 2020[1]. Therefore, using Ecoinvent datasets would result in underestimating impacts, particularly due to the lower NOx, CO, VOC, and particulate matter emissions; *(ii)* the generic datasets consider average load factors for transporting conventional goods. However, NH_3 tanker trucks usually perform outbound journeys with a full load and return empty, resulting in lower load factors and higher emissions per-tonne-kilometre (tkm); *(iii)* the estimated vehicle operation emissions of Ecoinvent datasets are based on older data (HBEFA 2010 and EMEP/EEA 2013) than the most recent EMEP/EAA guidebook[2]; and *(iv)* Ecoinvent used standardized European driving cycle tests, which differ from real Australian driving conditions, where most routes to rural areas single carriageways with sections that have particular high slopes or low speeds when crossing urban areas, resulting in varying driving conditions. Therefore, it was necessary to split the route into different sections.

For long and medium distances transport, average articulated and rigid trucks in Australia were used, respectively. The inventories for these trucks were modelled and are presented in Table S13 and Table S22. The shares among Euro technologies correspond to the percentages for each kind of vehicle in Australia presented in SAFC, 2020[1]. For the last step of the NH_3 distribution, the short distance performed in a van to deliver at farms, the Ecoinvent dataset for light commercial vehicles was used.

---

[1] SAFC (South Australian Freight Council). 2020. Profile of Australia's Trucking Fleet: Age and Emissions. Accessed: 06-April-2023. https://static1.squarespace.com/static/51297e5de4b01fa6748bc904/t/5a384f42f9619a424ba3300a/1513639758710/557_trucking_fleet_overview.pdf

[2] EEA (European Environment Agency) and EMEP (European Monitoring and Evaluation Programme). 2019. EMEP/EEA air pollutant emission inventory guidebook: Technical guidance to prepare national emission inventories. Luxembourg.

Articulated trucks

Table S13. LCI for Truck transport of liquid ammonia in 43.5-ton articulated truck **(Australian average)**

Products	Amount	unit
Truck transport liquid ammonia in 43.5-ton articulated truck {AU} AUSTRALIAN AVERAGE	1	tkm
**Avoided products**		
**Materials/fuels**		
Truck transport liquid ammonia in 43.5-ton articulated truck {AU} PRE-EURO	*0.214*	*tkm*
Truck transport liquid ammonia in 43.5-ton articulated truck {AU} EURO I	*0.180*	*tkm*
Truck transport liquid ammonia in 43.5-ton articulated truck {AU} EURO III	*0.293*	*tkm*
Truck transport liquid ammonia in 43.5-ton articulated truck {AU} EURO IV	*0.197*	*tkm*
Truck transport liquid ammonia in 43.5-ton articulated truck {AU} EURO V	*0.115*	*tkm*
**Electricity/heat**		
**Emissions to air**		
**Emissions to water**		
**Emissions to soil**		
**Waste to treatment**		

Table S14. LCI for Truck transport of liquid ammonia in 43.5-ton articulated truck {AU} (PRE-EURO to EURO V).
Note: This table represents each dataset included in Table S13.

Products	Amount	unit
Truck transport liquid ammonia in 43.5-ton articulated truck {AU} ***(PRE-EURO to EURO V)***	1	tkm
**Avoided products**		
**Materials/fuels**		
Lorry, 40 metric-ton {GLO}| market for	*1.07E-07*	*p*
Road {AU}| construction	*0.00148*	*my*
Diesel B5 {AU}| market group for	*0.0314*	*kg*
Transport (only traffic) in 43.5-ton articulated truck {AU} ***(PRE-EURO to EURO V)***	*1*	*tkm*
Urea solution (32.5%) {GLO}| market for ***(ONLY FOR VEHICLES EURO V)***	*3.06E-3*	*kg*
**Electricity/heat**		
**Emissions to air**		
**Emissions to water**		
**Emissions to soil**		
**Waste to treatment**		
Used lorry, 40 metric-ton {AU}| treatment of	*1.07E-07*	*p*
Decommissioned road {AU}| treatment of	*0.00148*	*my*

Table S15. LCI for Transport (only traffic) in 43.5-ton articulated truck {AU} **(PRE-EURO to EURO V)**

Note: This table represents the dataset *Transport (only traffic)* included in Table S14, for the different Euro technologies

**Products**	**Amount**	**unit**
Truck transport liquid ammonia in 43.5-ton articulated truck {AU} ***(PRE-EURO to EURO V)***	1	tkm
**Avoided products**		
**Materials/fuels**		
Maintenance, lorry 40 metric-ton {AU}| processing	*4.80E-07*	*p*
Road maintenance {AU}| road maintenance	*0.000284*	*my*
**Electricity/heat**		
**Emissions to air**		
**Emissions to water**		
**Emissions to soil**		
**Waste (grouped outputs)**		
Diesel combustion emissions per tkm 43.5-ton articulated truck *{AU}* ***(PRE-EURO to EURO V)***	*1*	*p (tkm)*
Engine oil consumption emissions per tkm 43.5-ton articulated truck *{AU}*	*1*	*p (tkm)*
Tyre abrasion emissions per tkm 43.5-ton articulated truck *{AU}*	*1*	*p (tkm)*
Brake abrasion emissions per tkm 43.5-ton articulated truck *{AU}*	*1*	*p (tkm)*
Road surface abrasion emissions per tkm 43.5-ton articulated truck *{AU}*	*1*	*p (tkm)*
Urea consumption emissions per tkm 43.5-ton articulated truck *{AU}* ***(ONLY FOR VEHICLES EURO V)***	*1*	*p (tkm)*

Table S16 corresponds to the LCI modelled for *Diesel combustion emissions per tkm* 43.5-ton articulated truck *{AU}* ***(PRE-EURO to EURO V)***, included in Table S15, which is the only output that varies depending on the Euro control emissions technology. The other outputs that depend on vehicle size, load, axles, speed, and road slopes, such as engine oil, urea, tyre, brake, and road surface abrasion emissions, are similar for every articulated truck regardless of the Euro technology. These LCI are presented in Tables S17-S21 for articulated trucks.

Table S16. LCI for Diesel combustion emissions per tkm 43.5-ton articulated truck {AU} (PRE-EURO to EURO V)

**Waste (grouped outputs)**			**Amount**			**unit**
Diesel combustion emissions per tkm 43.5-ton truck {AU}	PRE-EURO	EURO I	EURO III	EURO IV	EURO V	p (tkm)
**Avoided products**						
**Materials/fuels**						
**Electricity/heat**						
**Emissions to air**						
2-Propenal, 2-methyl-	*2.56E-07*	*2.34E-07*	*1.19E-07*	*2.52E-08*	*1.10E-08*	*kg*
Acetaldehyde	*1.30E-06*	*1.24E-06*	*6.35E-07*	*1.34E-07*	*5.83E-08*	*kg*
Acrolein	*5.05E-07*	*4.82E-07*	*2.46E-07*	*5.20E-08*	*2.26E-08*	*kg*
Aldehydes, C5	*2.57E-08*	*2.45E-08*	*1.25E-08*	*2.64E-09*	*1.15E-09*	*kg*
Alkanes, C13	*7.85E-06*	*7.48E-06*	*3.82E-06*	*8.07E-07*	*3.51E-07*	*kg*
Ammonia	*2.40E-07*	*2.40E-07*	*2.40E-07*	*2.40E-07*	*8.80E-07*	*kg*
Aromatics, C13	*5.82E-06*	*5.54E-06*	*2.83E-06*	*5.98E-07*	*2.60E-07*	*kg*
Aromatics, C9	*3.34E-07*	*3.18E-07*	*1.63E-07*	*3.44E-08*	*1.49E-08*	*kg*
Arsenic	*3.14E-12*	*3.14E-12*	*3.14E-12*	*3.14E-12*	*3.14E-12*	*kg*
Benzaldehyde	*3.91E-07*	*3.73E-07*	*1.90E-07*	*4.02E-08*	*1.75E-08*	*kg*
Benzaldehyde, 2-methyl-	*2.28E-07*	*2.18E-07*	*1.11E-07*	*2.35E-08*	*1.02E-08*	*kg*
Benzaldehyde, 3-methyl-	*1.68E-07*	*1.61E-07*	*8.20E-08*	*1.73E-08*	*7.53E-09*	*kg*
Benzene	*2.00E-08*	*1.90E-08*	*9.72E-09*	*2.06E-09*	*8.93E-10*	*kg*
Butadiene	*9.42E-07*	*8.98E-07*	*4.58E-07*	*9.69E-08*	*4.21E-08*	*kg*
Butanal	*2.51E-07*	*2.39E-07*	*1.22E-07*	*2.58E-08*	*1.12E-08*	*kg*
Butane	*4.28E-08*	*4.08E-08*	*2.08E-08*	*4.40E-09*	*1.91E-09*	*kg*
Cadmium	*1.57E-12*	*1.57E-12*	*1.57E-12*	*1.57E-12*	*1.57E-12*	*kg*
Carbon dioxide, fossil	*9.38E-02*	*9.38E-02*	*9.38E-02*	*9.38E-02*	*9.38E-02*	*kg*
Carbon monoxide, fossil	*1.71E-04*	*1.56E-04*	*1.44E-04*	*5.70E-05*	*1.13E-04*	*kg*
Chromium	*2.67E-10*	*2.67E-10*	*2.67E-10*	*2.67E-10*	*2.67E-10*	*kg*
Copper	*1.79E-10*	*1.79E-10*	*1.79E-10*	*1.79E-10*	*1.79E-10*	*kg*
Crotonaldehyde	*4.23E-07*	*4.03E-07*	*2.06E-07*	*4.35E-08*	*1.89E-08*	*kg*
Cycloalkanes, C16	*3.31E-07*	*3.16E-07*	*1.61E-07*	*3.41E-08*	*1.48E-08*	*kg*
Decane	*5.11E-07*	*4.87E-07*	*2.49E-07*	*5.26E-08*	*2.28E-08*	*kg*
Dibenzofuran	*4.30E-15*	*4.79E-15*	*4.77E-15*	*4.99E-15*	*5.85E-17*	*kg*

Dibenzo-p-dioxin	*2.83E-15*	*3.15E-15*	*3.14E-15*	*3.29E-15*	*4.03E-17*	*kg*
Dinitrogen monoxide	*2.40E-06*	*8.85E-07*	*5.61E-07*	*1.54E-06*	*4.46E-06*	*kg*
Ethane	*8.56E-09*	*8.16E-09*	*4.17E-09*	*8.81E-10*	*3.83E-10*	*kg*
Ethylene oxide	*2.00E-06*	*1.91E-06*	*9.74E-07*	*2.06E-07*	*8.95E-08*	*kg*
Ethyne	*3.00E-07*	*2.86E-07*	*1.46E-07*	*3.08E-08*	*1.34E-08*	*kg*
Formaldehyde	*2.40E-06*	*2.29E-06*	*1.17E-06*	*2.47E-07*	*1.07E-07*	*kg*
Heptane	*8.56E-08*	*8.16E-08*	*4.17E-08*	*8.81E-09*	*3.83E-09*	*kg*
Heptane, 2-methyl-	*5.99E-08*	*5.71E-08*	*2.92E-08*	*6.17E-09*	*2.68E-09*	*kg*
Heptane, 3-methyl-	*7.71E-08*	*7.35E-08*	*3.75E-08*	*7.93E-09*	*3.45E-09*	*kg*
Hexanal	*4.05E-07*	*3.86E-07*	*1.97E-07*	*4.71E-08*	*1.81E-08*	*kg*
Hexane, 2-methyl-	*1.80E-07*	*1.71E-07*	*8.75E-08*	*1.85E-08*	*8.04E-09*	*kg*
Hexane, 3-methyl-	*9.99E-08*	*9.52E-08*	*4.86E-08*	*1.03E-08*	*4.47E-09*	*kg*
Isobutane	*4.00E-08*	*3.81E-08*	*1.94E-08*	*4.11E-09*	*1.79E-09*	*kg*
Isobutene	*4.85E-07*	*4.62E-07*	*2.36E-07*	*4.99E-08*	*2.17E-08*	*kg*
Isobutyraldehyde	*1.68E-07*	*1.61E-07*	*8.20E-08*	*1.73E-08*	*7.53E-09*	*kg*
Lead	*1.57E-11*	*1.57E-11*	*1.57E-11*	*1.57E-11*	*1.57E-11*	*kg*
Mercury	*1.67E-10*	*1.67E-10*	*1.67E-10*	*1.67E-10*	*1.67E-10*	*kg*
Methane, fossil	*5.67E-06*	*5.67E-06*	*5.12E-06*	*3.37E-07*	*3.37E-07*	*kg*
m-Xylene	*2.80E-07*	*2.67E-07*	*1.36E-07*	*2.88E-08*	*1.25E-08*	*kg*
Nickel	*6.29E-13*	*6.29E-13*	*6.29E-13*	*6.29E-13*	*6.29E-13*	*kg*
Nitrogen dioxide	*1.02E-04*	*7.04E-05*	*7.53E-05*	*3.62E-05*	*1.56E-05*	*kg*
Nitrogen oxides	*8.27E-04*	*5.69E-04*	*4.63E-04*	*3.25E-04*	*1.14E-04*	*kg*
NMVOC, non-methane volatile organic compounds	*9.39E-07*	*8.95E-07*	*4.47E-07*	*9.66E-08*	*4.20E-08*	*kg*
o-Xylene	*1.14E-07*	*1.09E-07*	*5.56E-08*	*1.17E-08*	*5.11E-09*	*kg*
PAH, polycyclic aromatic hydrocarbons	*2.73E-08*	*3.04E-08*	*3.03E-08*	*3.17E-08*	*3.13E-08*	*kg*
Particulates, < 2.5 um	*3.06E-05*	*2.40E-05*	*1.11E-05*	*2.51E-06*	*2.94E-06*	*kg*
Pentanal	*1.14E-07*	*1.09E-07*	*5.56E-08*	*1.17E-08*	*5.11E-09*	*kg*
Pentane	*1.71E-08*	*1.63E-08*	*8.34E-09*	*1.76E-09*	*7.66E-10*	*kg*
Polychlorinated biphenyls	*1.26E-15*	*1.59E-15*	*1.58E-15*	*1.66E-15*	*1.95E-17*	*kg*
Propanal	*3.57E-07*	*3.40E-07*	*1.74E-07*	*3.67E-08*	*1.60E-08*	*kg*
Propane	*2.85E-08*	*2.72E-08*	*1.39E-08*	*2.94E-09*	*1.28E-09*	*kg*
Propylene glycol	*3.77E-07*	*3.59E-07*	*1.83E-07*	*3.88E-08*	*1.68E-08*	*kg*
Selenium	*3.14E-13*	*3.14E-13*	*3.14E-13*	*3.14E-13*	*3.14E-13*	*kg*
Styrene	*1.60E-07*	*1.52E-07*	*7.78E-08*	*1.64E-08*	*7.15E-09*	*kg*
Sulfur dioxide	*6.29E-07*	*6.29E-07*	*6.39E-07*	*6.29E-07*	*6.29E-07*	*kg*
Toluene	*2.85E-09*	*2.72E-09*	*1.39E-09*	*2.94E-10*	*1.28E-10*	*kg*
Trimethylbenzenes	*4.60E-07*	*4.38E-07*	*2.24E-07*	*4.73E-08*	*2.05E-08*	*kg*
Zinc	*5.66E-10*	*5.66E-10*	*5.66E-10*	*5.66E-10*	*5.66E-10*	*kg*
**Emissions to water**						
**Emissions to soil**						
**Waste treatment**						

Table S17. LCI for Engine oil consumption emissions per tkm 43.5-ton articulated truck {AU}

**Waste (grouped outputs)**	**Amount**	**unit**
Engine oil consumption emissions per tkm 43.5-ton articulated truck {AU}	1	p (tkm)
**Avoided products**		
**Materials/fuels**		
**Electricity/heat**		
**Emissions to air**		
Cadmium	*5.69E-11*	*kg*
Carbon dioxide, fossil	*3.89E-05*	*kg*
Chromium	*2.40E-10*	*kg*
Copper	*9.71E-09*	*kg*
Lead	*4.14E-13*	*kg*
Nickel	*3.98E-10*	*kg*
Selenium	*5.67E-11*	*kg*
Zinc	*5.62E-09*	*kg*
**Emissions to water**		
**Emissions to soil**		
**Waste treatment**		

Table S18. LCI for Tyre abrasion emissions per tkm 43.5-ton articulated truck {AU}

**Waste (grouped outputs)**	**Amount**	**unit**
Tyre abrasion emissions per tkm 43.5-ton articulated truck {AU}	1	p (tkm)
**Avoided products**		
**Materials/fuels**		
**Electricity/heat**		
**Emissions to air**		
Aluminium	*2.22E-10*	*kg*
Ammonium, ion	*1.30E-10*	*kg*
Antimony	*1.37E-12*	*kg*
Arsenic	*2.60E-12*	*kg*
Barium	*8.57E-11*	*kg*
Benzo(a)pyrene	*2.67E-12*	*kg*
Bromine	*1.37E-11*	*kg*
Cadmium	*3.22E-12*	*kg*
Calcium	*6.11E-10*	*kg*
Chloride	*4.11E-10*	*kg*
Chlorine	*3.56E-10*	*kg*
Chromium	*1.63E-11*	*kg*
Cobalt	*8.77E-12*	*kg*
Copper	*1.19E-12*	*kg*
Iron	*1.17E-09*	*kg*
Lead	*1.21E-10*	*kg*
Lithium	*8.91E-13*	*kg*
Magnesium	*1.14E-10*	*kg*
Manganese	*3.50E-11*	*kg*
Molybdenum	*1.92E-12*	*kg*
Nickel	*2.50E-11*	*kg*
Nitrate	*1.03E-09*	*kg*
Particulates, < 2.5 um	*2.06E-06*	*kg*
Particulates, > 10 um	*1.96E-06*	*kg*
Particulates, > 2.5 um, and < 10um	*8.81E-07*	*kg*
Potassium	*1.92E-10*	*kg*
Selenium	*1.37E-11*	*kg*
Silicon	*1.23E-09*	*kg*
Silver	*6.85E-14*	*kg*
Sodium	*4.42E-10*	*kg*
Strontium	*9.87E-12*	*kg*
Sulfate	*1.71E-09*	*kg*
Sulfur dioxide	*7.54E-10*	*kg*
Titanium	*2.59E-10*	*kg*
Vanadium	*6.85E-13*	*kg*
Zinc	*5.10E-09*	*kg*
**Emissions to water** *(groundwater)*		
Aluminium	*6.82E-10*	*kg*
Ammonium, ion	*4.00E-10*	*kg*
Antimony	*4.21E-12*	*kg*
Arsenic	*8.00E-12*	*kg*
Barium	*2.63E-10*	*kg*
Benzo(a)pyrene	*8.21E-12*	*kg*
Bromine	*4.21E-11*	*kg*
Cadmium	*9.90E-12*	*kg*
Calcium	*1.88E-09*	*kg*
Chloride	*1.26E-09*	*kg*
Chlorine	*1.09E-09*	*kg*
Chromium	*5.01E-11*	*kg*
Cobalt	*2.69E-11*	*kg*
Copper	*3.66E-10*	*kg*
Iron	*3.60E-09*	*kg*
Lead	*3.71E-10*	*kg*
Lithium	*2.74E-12*	*kg*
Magnesium	*3.49E-10*	*kg*

Manganese	*1.07E-10*	*kg*
Molybdenum	*5.90E-12*	*kg*
Nickel	*6.30E-11*	*kg*
Nitrate	*3.10E-09*	*kg*
Potassium	*5.90E-10*	*kg*
Selenium	*4.21E-11*	*kg*
Silicon	*3.79E-09*	*kg*
Silver	*2.11E-13*	*kg*
Sodium	*1.36E-09*	*kg*
Strontium	*3.03E-11*	*kg*
Sulfate	*5.26E-09*	*kg*
Sulfur	*2.32E-09*	*kg*
Titanium	*7.96E-10*	*kg*
Vanadium	*2.11E-12*	*kg*
Zinc	*1.57E-08*	*kg*
**Emissions to soil**		
Aluminium	*6.82E-10*	*kg*
Ammonium, ion	*4.00E-10*	*kg*
Antimony	*4.21E-12*	*kg*
Arsenic	*8.00E-12*	*kg*
Barium	*2.63E-10*	*kg*
Benzo(a)pyrene	*8.21E-12*	*kg*
Bromine	*4.21E-11*	*kg*
Cadmium	*9.90E-12*	*kg*
Calcium	*1.88E-09*	*kg*
Chloride	*1.26E-09*	*kg*
Chlorine	*1.09E-09*	*kg*
Chromium	*5.01E-11*	*kg*
Cobalt	*2.69E-11*	*kg*
Copper	*3.66E-10*	*kg*
Iron	*3.60E-09*	*kg*
Lead	*3.71E-10*	*kg*
Lithium	*2.74E-12*	*kg*
Magnesium	*3.49E-10*	*kg*
Manganese	*1.07E-10*	*kg*
Molybdenum	*5.90E-12*	*kg*
Nickel	*6.30E-11*	*kg*
Nitrate	*3.12E-09*	*kg*
Potassium	*5.90E-10*	*kg*
Selenium	*4.21E-11*	*kg*
Silicon	*3.79E-09*	*kg*
Silver	*2.11E-13*	*kg*
Sodium	*1.36E-09*	*kg*
Strontium	*3.03E-11*	*kg*
Sulfate	*5.26E-09*	*kg*
Sulfur	*2.32E-09*	*kg*
Titanium	*7.96E-10*	*kg*
Vanadium	*2.11E-12*	*kg*
Zinc	*1.57E-08*	*kg*
**Waste treatment**		

Table S19. LCI for Brake abrasion emissions per tkm 43.5-ton articulated truck {AU}

**Waste (grouped outputs)**	**Amount**	**unit**
Brake abrasion emissions per tkm 43.5-ton articulated truck {AU}	1	p (tkm)
**Avoided products**		
**Materials/fuels**		
**Electricity/heat**		
**Emissions to air**		
Aluminium	*1.25E-09*	*kg*
Ammonium, ion	*1.84E-11*	*kg*
Antimony	*6.12E-09*	*kg*

Arsenic	*4.13E-11*	*kg*
Barium	*2.36E-08*	*kg*
Benzo(a)pyrene	*4.53E-13*	*kg*
Benzo(b)fluoranthene	*2.57E-13*	*kg*
Benzo(k)fluoranthene	*3.79E-13*	*kg*
Bromine	*2.45E-11*	*kg*
Cadmium	*1.37E-11*	*kg*
Calcium	*4.71E-10*	*kg*
Chloride	*9.18E-10*	*kg*
Chlorine	*9.18E-10*	*kg*
Chromium	*1.41E-09*	*kg*
Cobalt	*3.92E-12*	*kg*
Copper	*3.13E-08*	*kg*
Iron	*1.28E-07*	*kg*
Lead	*3.75E-09*	*kg*
Lithium	*3.40E-11*	*kg*
Magnesium	*2.73E-08*	*kg*
Manganese	*1.51E-09*	*kg*
Molybdenum	*6.12E-09*	*kg*
Nickel	*2.00E-10*	*kg*
Nitrate	*9.79E-10*	*kg*
Particulates, < 2.5 um	*2.39E-07*	*kg*
Particulates, > 10 um	*1.22E-08*	*kg*
Particulates, > 2.5 um, and < 10um	*3.61E-07*	*kg*
Potassium	*3.20E-10*	*kg*
Potassium	*3.06E-11*	*kg*
Selenium	*1.22E-11*	*kg*
Silicon	*4.16E-08*	*kg*
Sodium	*4.74E-09*	*kg*
Strontium	*3.18E-10*	*kg*
Sulfate	*2.04E-08*	*kg*
Sulfur	*7.83E-09*	*kg*
Tin	*4.28E-09*	*kg*
Titanium	*2.20E-09*	*kg*
Vanadium	*4.04E-10*	*kg*
Zinc	*5.31E-09*	*kg*
**Emissions to water**		
**Emissions to soil**		
**Waste treatment**		

Table S20. LCI for Road surface abrasion emissions per tkm 43.5-ton articulated truck {AU}

**Waste (grouped outputs)**	**Amount**	**unit**
Road surface abrasion emissions per tkm 43.5-ton articulated truck {AU}	1	p (tkm)
**Avoided products**		
**Materials/fuels**		
**Electricity/heat**		
**Emissions to air**		
Particulates, < 2.5 um	*1.64E-06*	*kg*
Particulates, > 10 um	*3.04E-06*	*kg*
Particulates, > 2.5 um, and < 10um	*1.40E-06*	*kg*
**Emissions to water**		
**Emissions to soil**		
**Waste treatment**		

Table S21. LCI for Urea consumption emissions per tkm 43.5-ton articulated truck {AU} (ONLY FOR EURO V)

Waste (grouped outputs)	Amount	unit
Urea consumption emissions per tkm 43.5-ton articulated truck {AU} ***(ONLY FOR EURO V)***	1	p (tkm)
**Avoided products**		
**Materials/fuels**		
**Electricity/heat**		
**Emissions to air**		
Carbon dioxide, fossil	*4.49E-04*	*kg*
**Emissions to water**		
**Emissions to soil**		
**Waste treatment**		

Rigid trucks

Table S22. LCI for Truck transport of liquid ammonia in 15-ton rigid truck **(Australian average)**

Products	Amount	unit
Truck transport liquid ammonia in 15-ton rigid truck {AU} AUSTRALIAN AVERAGE	1	tkm
**Avoided products**		
**Materials/fuels**		
Truck transport liquid ammonia in 15-ton rigid truck {AU} PRE-EURO	*0.351*	*tkm*
Truck transport liquid ammonia in 15-ton rigid truck {AU} EURO I	*0.185*	*tkm*
Truck transport liquid ammonia in 15-ton rigid truck {AU} EURO III	*0.219*	*tkm*
Truck transport liquid ammonia in 15-ton rigid truck {AU} EURO IV	*0.154*	*tkm*
Truck transport liquid ammonia in 15-ton rigid truck {AU} EURO V	*0.090*	*tkm*
**Electricity/heat**		
**Emissions to air**		
**Emissions to water**		
**Emissions to soil**		
**Waste to treatment**		

Table S23. LCI for Truck transport of liquid ammonia in 15-ton rigid truck {AU} (PRE-EURO to EURO V).
Note: This table represents each dataset included in Table S22.

Products	Amount	unit
Truck transport liquid ammonia in 15-ton rigid truck {AU} ***(PRE-EURO to EURO V)***	1	tkm
**Avoided products**		
**Materials/fuels**		
Lorry, 16 metric-ton {GLO}| market for	*3.81E-7*	*p*
Road {AU}| construction	*1.96E-3*	*my*
Diesel B5 {AU}| market group for	*5.50E-2*	*kg*
Transport (only traffic) in 15-ton rigid truck {AU} ***(PRE-EURO to EURO V)***	*1*	*tkm*
Urea solution (32.5%) {GLO}| market for ***(ONLY FOR VEHICLES EURO V)***	*4.09E-3*	*kg*
**Electricity/heat**		
**Emissions to air**		
**Emissions to water**		
**Emissions to soil**		
**Waste to treatment**		
Used lorry, 16 metric-ton {AU}| treatment of	*3.81E-7*	*p*
Decommissioned road {AU}| treatment of	*1.96E-3*	*my*

Table S24. LCI for Transport (only traffic) in 15-ton rigid truck {AU} **(PRE-EURO to EURO V)**

Note: This table represents the dataset *Transport (only traffic)* included in Table S23, for the different Euro technologies

**Products**	**Amount**	**unit**
Truck transport liquid ammonia in 15-ton rigid truck {AU} ***(PRE-EURO to EURO V)***	1	tkm
**Avoided products**		
**Materials/fuels**		
Maintenance, lorry 16 metric-ton {AU}| processing	*2.86E-6*	*p*
Road maintenance {AU}| road maintenance	*1.02E-3*	*my*
**Electricity/heat**		
**Emissions to air**		
**Emissions to water**		
**Emissions to soil**		
**Waste (grouped outputs)**		
Diesel combustion emissions per tkm 15-ton rigid truck *{AU}* ***(PRE-EURO to EURO V)***	*1*	*p (tkm)*
Engine oil consumption emissions per tkm 15-ton rigid truck *{AU}*	*1*	*p (tkm)*
Tyre abrasion emissions per tkm 15-ton rigid truck *{AU}*	*1*	*p (tkm)*
Brake abrasion emissions per tkm 15-ton rigid truck *{AU}*	*1*	*p (tkm)*
Road surface abrasion emissions per tkm 15-ton rigid truck *{AU}*	*1*	*p (tkm)*
Urea consumption emissions per tkm 15-ton rigid truck *{AU}* ***(ONLY FOR VEHICLES EURO V)***	*1*	*p (tkm)*

Table S25 corresponds to the LCI modelled for *Diesel combustion emissions per tkm* 15-ton rigid truck *{AU}* ***(PRE-EURO to EURO V)***, included in Table S24, which is the only output that varies depending on the Euro control emissions technology. The other outputs that depend on vehicle size, load, axles, speed, and road slopes, such as engine oil, urea, tyre, brake, and road surface abrasion emissions, are similar for every rigid truck regardless of the Euro technology. These LCI are presented in Tables S26-S30 for rigid trucks.

Table S25. LCI for Diesel combustion emissions per tkm 15-ton rigid truck {AU} (PRE-EURO to EURO V)

**Waste (grouped outputs)**			**Amount**			**unit**
Diesel combustion emissions per tkm 15-ton truck {AU}	PRE-EURO	EURO I	EURO III	EURO IV	EURO V	p (tkm)
**Avoided products**						
**Materials/fuels**						
**Electricity/heat**						
**Emissions to air**						
2-Propenal, 2-methyl-	*1.68E-06*	*6.72E-07*	*3.89E-07*	*6.16E-08*	*3.04E-08*	*kg*
Acetaldehyde	*8.90E-06*	*3.57E-06*	*2.07E-06*	*3.28E-07*	*1.61E-07*	*kg*
Acrolein	*3.45E-06*	*1.38E-06*	*8.01E-06*	*1.27E-07*	*6.25E-08*	*kg*
Aldehydes, C5	*1.77E-07*	*7.03E-08*	*4.07E-08*	*6.45E-09*	*3.18E-09*	*kg*
Alkanes, C13	*5.36E-05*	*2.15E-05*	*1.24E-05*	*1.97E-06*	*9.71E-07*	*kg*
Ammonia	*8.57E-07*	*8.57E-07*	*8.57E-07*	*8.57E-07*	*3.14E-06*	*kg*
Aromatics, C13	*3.97E-05*	*1.59E-05*	*9.21E-06*	*1.45E-06*	*7.19E-07*	*kg*
Aromatics, C9	*2.28E-06*	*9.14E-07*	*5.29E-07*	*8.39E-08*	*4.13E-08*	*kg*
Arsenic	*5.50E-12*	*5.50E-12*	*5.50E-12*	*5.50E-12*	*5.50E-12*	*kg*
Benzaldehyde	*2.67E-06*	*1.07E-06*	*6.20E-07*	*9.82E-08*	*4.84E-08*	*kg*
Benzaldehyde, 2-methyl-	*1.56E-06*	*6.25E-07*	*3.62E-07*	*5.73E-08*	*2.82E-08*	*kg*
Benzaldehyde, 3-methyl-	*1.15E-06*	*4.61E-07*	*2.67E-07*	*4.23E-08*	*2.08E-08*	*kg*
Benzene	*1.36E-07*	*5.47E-08*	*3.17E-08*	*5.02E-09*	*2.47E-09*	*kg*
Butadiene	*6.43E-06*	*2.58E-06*	*1.49E-06*	*2.37E-07*	*1.17E-07*	*kg*
Butanal	*1.71E-06*	*6.87E-07*	*3.98E-07*	*6.31E-08*	*3.11E-08*	*kg*
Butane	*2.92E-07*	*1.17E-07*	*6.79E-08*	*1.08E-08*	*5.30E-09*	*kg*
Cadmium	*2.75E-12*	*2.75E-12*	*2.75E-12*	*2.75E-12*	*2.75E-12*	*kg*
Carbon dioxide, fossil	*1.64E-01*	*1.64E-01*	*1.64E-01*	*1.64E-01*	*1.64E-01*	*kg*
Carbon monoxide, fossil	*6.17E-04*	*3.18E-04*	*3.02E-04*	*1.45E-04*	*2.73E-04*	*kg*
Chromium	*4.67E-10*	*4.67E-10*	*4.97E-10*	*4.67E-10*	*4.67E-10*	*kg*
Copper	*3.13E-10*	*3.13E-10*	*3.13E-10*	*3.13E-10*	*3.13E-10*	*kg*
Crotonaldehyde	*2.88E-06*	*1.16E-06*	*6.70E-07*	*1.06E-07*	*5.23E-08*	*kg*
Cycloalkanes, C16	*2.26E-06*	*9.06E-07*	*5.25E-07*	*8.31E-08*	*4.10E-08*	*kg*

Decane	*3.49E-06*	*1.40E-06*	*8.10E-07*	*1.28E-07*	*6.32E-08*	*kg*
Dibenzofuran	*1.08E-14*	*1.27E-14*	*1.26E-14*	*1.26E-14*	*1.60E-16*	*kg*
Dibenzo-p-dioxin	*7.09E-15*	*8.33E-15*	*8.31E-15*	*8.31E-15*	*1.10E-16*	*kg*
Dinitrogen monoxide	*8.57E-06*	*2.01E-06*	*1.15E-06*	*3.26E-06*	*9.59E-06*	*kg*
Ethane	*5.85E-08*	*2.34E-08*	*1.36E-08*	*2.15E-09*	*1.06E-09*	*kg*
Ethylene oxide	*1.37E-05*	*5.48E-06*	*3.17E-06*	*5.02E-07*	*2.48E-07*	*kg*
Ethyne	*2.05E-06*	*8.20E-07*	*4.75E-07*	*7.53E-08*	*3.71E-08*	*kg*
Formaldehyde	*1.64E-05*	*6.56E-06*	*3.80E-06*	*6.02E-07*	*2.97E-07*	*kg*
Heptane	*5.85E-07*	*2.34E-07*	*1.36E-07*	*2.15E-08*	*1.06E-08*	*kg*
Heptane, 2-methyl-	*4.09E-07*	*1.64E-07*	*9.50E-08*	*1.51E-08*	*7.41E-09*	*kg*
Heptane, 3-methyl-	*5.26E-07*	*2.11E-07*	*1.22E-07*	*1.94E-08*	*9.53E-09*	*kg*
Hexanal	*2.77E-06*	*1.11E-06*	*6.42E-07*	*1.02E-07*	*5.01E-08*	*kg*
Hexane, 2-methyl-	*1.23E-06*	*4.92E-07*	*2.85E-07*	*4.52E-08*	*2.22E-08*	*kg*
Hexane, 3-methyl-	*6.82E-07*	*2.73E-07*	*1.58E-07*	*2.51E-08*	*1.24E-08*	*kg*
Isobutane	*2.73E-07*	*1.09E-07*	*6.33E-08*	*1.00E-08*	*4.94E-09*	*kg*
Isobutene	*3.31E-06*	*1.33E-06*	*7.69E-07*	*1.22E-07*	*6.00E-08*	*kg*
Isobutyraldehyde	*1.15E-06*	*4.61E-07*	*2.67E-07*	*4.23E-08*	*2.08E-08*	*kg*
Lead	*2.75E-11*	*2.75E-11*	*2.75E-11*	*2.75E-11*	*2.75E-11*	*kg*
Mercury	*2.91E-10*	*2.91E-10*	*2.91E-10*	*2.91E-10*	*2.91E-10*	*kg*
Methane, fossil	*5.87E-06*	*5.87E-06*	*5.27E-06*	*3.46E-07*	*3.46E-07*	*kg*
m-Xylene	*1.91E-06*	*7.65E-07*	*4.43E-07*	*7.02E-08*	*3.46E-08*	*kg*
Nickel	*1.10E-12*	*1.10E-12*	*1.10E-12*	*1.10E-12*	*1.10E-12*	*kg*
Nitrogen dioxide	*2.91E-04*	*1.72E-04*	*1.76E-04*	*9.05E-05*	*3.67E-05*	*kg*
Nitrogen oxides	*2.35E-03*	*1.39E-03*	*1.08E-03*	*8.14E-04*	*2.69E-04*	*kg*
NMVOC, non-methane volatile organic compounds	*6.41E-06*	*2.57E-06*	*1.49E-06*	*2.36E-07*	*1.16E-07*	*kg*
o-Xylene	*7.79E-07*	*3.12E-07*	*1.81E-07*	*2.87E-08*	*1.41E-08*	*kg*
PAH, polycyclic aromatic hydrocarbons	*6.84E-08*	*8.03E-08*	*8.02E-08*	*8.02E-08*	*8.55E-08*	*kg*
Particulates, < 2.5 um	*8.04E-05*	*4.84E-05*	*2.41E-05*	*6.25E-06*	*7.51E-06*	*kg*
Pentanal	*7.79E-07*	*3.12E-07*	*1.81E-07*	*2.87E-08*	*1.41E-08*	*kg*
Pentane	*1.17E-07*	*4.69E-08*	*2.71E-08*	*4.30E-09*	*2.12E-09*	*kg*
Polychlorinated biphenyls	*3.09E-15*	*4.20E-15*	*4.19E-15*	*4.19E-15*	*5.32E-17*	*kg*
Propanal	*2.44E-06*	*9.76E-07*	*5.65E-07*	*8.96E-08*	*4.41E-08*	*kg*
Propane	*1.95E-07*	*7.81E-08*	*4.52E-08*	*7.17E-09*	*3.53E-09*	*kg*
Propylene glycol	*2.57E-06*	*1.03E-06*	*5.97E-07*	*9.46E-08*	*4.66E-08*	*kg*
Selenium	*5.50E-13*	*5.50E-13*	*5.50E-13*	*5.50E-13*	*5.50E-13*	*kg*
Styrene	*1.09E-06*	*4.37E-07*	*2.53E-07*	*4.01E-08*	*1.98E-08*	*kg*
Sulfur dioxide	*1.10E-06*	*1.10E-06*	*1.10E-06*	*1.10E-06*	*1.10E-06*	*kg*
Toluene	*1.95E-08*	*7.81E-09*	*4.52E-09*	*7.17E-10*	*3.53E-10*	*kg*
Trimethylbenzenes	*3.14E-06*	*1.26E-06*	*7.28E-07*	*1.15E-07*	*5.68E-08*	*kg*
Zinc	*9.89E-10*	*9.89E-10*	*9.89E-10*	*9.89E-10*	*9.89E-10*	*kg*
**Emissions to water**						
**Emissions to soil**						
**Waste treatment**						

Table S26. LCI for Engine oil consumption emissions per tkm 15-ton rigid truck {AU}

**Waste (grouped outputs)**	**Amount**	**unit**
Engine oil consumption emissions per tkm 15-ton rigid truck {AU}	1	p (tkm)
**Avoided products**		
**Materials/fuels**		
**Electricity/heat**		
**Emissions to air**		
Cadmium	*2.03E-10*	*kg*
Carbon dioxide, fossil	*1.39E-04*	*kg*
Chromium	*8.56E-10*	*kg*
Copper	*3.47E-08*	*kg*
Lead	*1.48E-12*	*kg*
Nickel	*1.42E-09*	*kg*
Selenium	*2.02E-10*	*kg*
Zinc	*2.01E-08*	*kg*
**Emissions to water**		
**Emissions to soil**		
**Waste treatment**		

Table S27. LCI for Tyre abrasion emissions per tkm 15-ton rigid truck {AU}

Waste (grouped outputs)	Amount	unit
Tyre abrasion emissions per tkm 15-ton rigid truck {AU}	1	p (tkm)
**Avoided products**		
**Materials/fuels**		
**Electricity/heat**		
**Emissions to air**		
Aluminium	*2.64E-10*	*kg*
Ammonium, ion	*1.55E-10*	*kg*
Antimony	*1.63E-12*	*kg*
Arsenic	*3.10E-12*	*kg*
Barium	*1.02E-10*	*kg*
Benzo(a)pyrene	*3.18E-12*	*kg*
Bromine	*1.63E-11*	*kg*
Cadmium	*3.84E-12*	*kg*
Calcium	*7.28E-10*	*kg*
Chloride	*4.90E-10*	*kg*
Chlorine	*4.24E-10*	*kg*
Chromium	*1.94E-11*	*kg*
Cobalt	*1.04E-11*	*kg*
Copper	*1.42E-10*	*kg*
Iron	*1.40E-09*	*kg*
Lead	*1.44E-10*	*kg*
Lithium	*1.06E-12*	*kg*
Magnesium	*1.35E-10*	*kg*
Manganese	*4.16E-11*	*kg*
Molybdenum	*2.28E-12*	*kg*
Nickel	*2.44E-11*	*kg*
Nitrate	*1.22E-09*	*kg*
Particulates, < 2.5 um	*2.45E-06*	*kg*
Particulates, > 10 um	*2.33E-06*	*kg*
Particulates, > 2.5 um, and < 10um	*1.05E-06*	*kg*
Potassium	*2.28E-10*	*kg*
Selenium	*1.63E-11*	*kg*
Silicon	*1.47E-09*	*kg*
Silver	*8.16E-14*	*kg*
Sodium	*5.26E-10*	*kg*
Strontium	*1.18E-11*	*kg*
Sulfate	*2.04E-09*	*kg*
Sulfur dioxide	*8.98E-10*	*kg*
Titanium	*3.08E-10*	*kg*
Vanadium	*8.16E-13*	*kg*
Zinc	*6.07E-09*	*kg*
**Emissions to water** *(groundwater)*		
Aluminium	*8.12E-10*	*kg*
Ammonium, ion	*4.76E-10*	*kg*
Antimony	*5.01E-12*	*kg*
Arsenic	*9.52E-12*	*kg*
Barium	*3.13E-10*	*kg*
Benzo(a)pyrene	*9.78E-12*	*kg*
Bromine	*5.01E-11*	*kg*
Cadmium	*1.18E-11*	*kg*
Calcium	*2.24E-09*	*kg*
Chloride	*1.50E-09*	*kg*
Chlorine	*1.30E-09*	*kg*
Chromium	*5.97E-11*	*kg*
Cobalt	*3.21E-11*	*kg*
Copper	*4.36E-10*	*kg*
Iron	*4.29E-09*	*kg*
Lead	*4.41E-10*	*kg*
Lithium	*3.26E-12*	*kg*
Magnesium	*4.16E-10*	*kg*
Manganese	*1.28E-10*	*kg*
Molybdenum	*7.02E-12*	*kg*

Nickel	*7.49E-11*	*kg*
Nitrate	*3.76E-09*	*kg*
Potassium	*7.02E-10*	*kg*
Selenium	*5.01E-11*	*kg*
Silicon	*4.51E-09*	*kg*
Silver	*2.51E-13*	*kg*
Sodium	*1.62E-09*	*kg*
Strontium	*3.61E-11*	*kg*
Sulfate	*6.27E-09*	*kg*
Sulfur	*2.76E-09*	*kg*
Titanium	*9.47E-10*	*kg*
Vanadium	*2.51E-12*	*kg*
Zinc	*1.86E-08*	*kg*
**Emissions to soil**		
Aluminium	*8.12E-10*	*kg*
Ammonium, ion	*4.76E-10*	*kg*
Antimony	*5.01E-12*	*kg*
Arsenic	*9.52E-12*	*kg*
Barium	*3.13E-10*	*kg*
Benzo(a)pyrene	*9.78E-12*	*kg*
Bromine	*5.01E-11*	*kg*
Cadmium	*1.18E-11*	*kg*
Calcium	*2.24E-09*	*kg*
Chloride	*1.50E-09*	*kg*
Chlorine	*1.30E-09*	*kg*
Chromium	*5.97E-11*	*kg*
Cobalt	*3.21E-11*	*kg*
Copper	*4.36E-10*	*kg*
Iron	*4.29E-09*	*kg*
Lead	*4.41E-10*	*kg*
Lithium	*3.26E-12*	*kg*
Magnesium	*4.16E-10*	*kg*
Manganese	*1.28E-10*	*kg*
Molybdenum	*7.02E-12*	*kg*
Nickel	*7.49E-11*	*kg*
Nitrate	*3.76E-09*	*kg*
Potassium	*7.02E-10*	*kg*
Selenium	*5.01E-11*	*kg*
Silicon	*4.51E-09*	*kg*
Silver	*2.51E-13*	*kg*
Sodium	*1.62E-09*	*kg*
Strontium	*3.61E-11*	*kg*
Sulfate	*6.27E-09*	*kg*
Sulfur	*2.76E-09*	*kg*
Titanium	*9.47E-10*	*kg*
Vanadium	*2.51E-12*	*kg*
Zinc	*1.86E-08*	*kg*
**Waste treatment**		

Table S28. LCI for Brake abrasion emissions per tkm 15-ton rigid truck {AU}

**Waste (grouped outputs)**	**Amount**	**unit**
Brake abrasion emissions per tkm 15-ton rigid truck {AU}	1	p (tkm)
**Avoided products**		
**Materials/fuels**		
**Electricity/heat**		
**Emissions to air**		
Aluminium	*4.48E-09*	*kg*
Ammonium, ion	*6.56E-11*	*kg*
Antimony	*2.19E-08*	*kg*
Arsenic	*1.48E-10*	*kg*
Barium	*8.42E-08*	*kg*
Benzo(a)pyrene	*1.62E-12*	*kg*
Benzo(b)fluoranthene	*9.18E-13*	*kg*
Benzo(k)fluoranthene	*1.36E-12*	*kg*

Bromine	*8.74E-11*	*kg*
Cadmium	*4.90E-11*	*kg*
Calcium	*1.68E-09*	*kg*
Chloride	*3.28E-09*	*kg*
Chlorine	*3.28E-09*	*kg*
Chromium	*5.05E-09*	*kg*
Cobalt	*1.40E-11*	*kg*
Copper	*1.12E-07*	*kg*
Iron	*4.58E-07*	*kg*
Lead	*1.33E-08*	*kg*
Lithium	*1.22E-10*	*kg*
Magnesium	*9.74E-08*	*kg*
Manganese	*5.38E-09*	*kg*
Molybdenum	*2.19E-08*	*kg*
Nickel	*7.15E-10*	*kg*
Nitrate	*3.50E-09*	*kg*
Particulates, < 2.5 um	*8.52E-07*	*kg*
Particulates, > 10 um	*4.37E-08*	*kg*
Particulates, > 2.5 um, and < 10um	*1.29E-06*	*kg*
Potassium	*1.14E-09*	*kg*
Potassium	*1.09E-10*	*kg*
Selenium	*4.37E-11*	*kg*
Silicon	*1.48E-07*	*kg*
Sodium	*1.69E-08*	*kg*
Strontium	*1.14E-09*	*kg*
Sulfate	*7.30E-08*	*kg*
Sulfur	*2.80E-08*	*kg*
Tin	*1.53E-08*	*kg*
Titanium	*7.87E-09*	*kg*
Vanadium	*1.44E-09*	*kg*
Zinc	*1.90E-08*	*kg*
**Emissions to water**		
**Emissions to soil**		
**Waste treatment**		

Table S29. LCI for Road surface abrasion emissions per tkm 15-ton rigid truck {AU}

**Waste (grouped outputs)**	**Amount**	**unit**
Road surface abrasion emissions per tkm 15-ton rigid truck {AU}	1	p (tkm)
**Avoided products**		
**Materials/fuels**		
**Electricity/heat**		
**Emissions to air**		
Particulates, < 2.5 um	*5.86E-06*	*kg*
Particulates, > 10 um	*1.09E-05*	*kg*
Particulates, > 2.5 um, and < 10um	*4.99E-06*	*kg*
**Emissions to water**		
**Emissions to soil**		
**Waste treatment**		

Table S30. LCI for Urea consumption emissions per tkm 15-ton rigid truck {AU} (ONLY FOR EURO V)

**Waste (grouped outputs)**	**Amount**	**unit**
Urea consumption emissions per tkm 15-ton rigid truck {AU} ***(ONLY FOR EURO V)***	1	p (tkm)
**Avoided products**		
**Materials/fuels**		
**Electricity/heat**		
**Emissions to air**		
Carbon dioxide, fossil	*7.85E-04*	*kg*
**Emissions to water**		
**Emissions to soil**		
**Waste treatment**		
